# A Two-Color Haploid Genetic Screen Identifies Novel Host Factors Involved in HIV-1 Latency

**DOI:** 10.1128/mBio.02980-21

**Published:** 2021-12-07

**Authors:** Michael Röling, Mahsa Mollapour Sisakht, Enrico Ne, Panagiotis Moulos, Raquel Crespo, Mateusz Stoszko, Elisa De Crignis, Helen Bodmer, Tsung Wai Kan, Maryam Akbarzadeh, Vaggelis Harokopos, Pantelis Hatzis, Robert-Jan Palstra, Tokameh Mahmoudi

**Affiliations:** a Department of Biochemistry, Erasmus University Medical Center, Rotterdam, the Netherlands; b Stem Cell and Regenerative Medicine Center of Excellence, Tehran University of Medical Sciences, Tehran, Iran; c Institute for Fundamental Biomedical Research, Biomedical Sciences Research Center Alexander Fleming, Vari, Greece; d Department of Urology, Erasmus University Medical Center, Rotterdam, the Netherlands; e Department of Pathology, Erasmus University Medical Center, Rotterdam, the Netherlands; University of Pittsburgh; University of Pittsburgh School of Medicine

**Keywords:** latency, haploid forward genetic screen, host factor, human immunodeficiency virus

## Abstract

To identify novel host factors as putative targets to reverse HIV-1 latency, we performed an insertional mutagenesis genetic screen in a latent HIV-1 infected pseudohaploid KBM7 cell line (Hap-Lat). Following mutagenesis, insertions were mapped to the genome, and bioinformatic analysis resulted in the identification of 69 candidate host genes involved in maintaining HIV-1 latency. A select set of candidate genes was functionally validated using short hairpin RNA (shRNA)-mediated depletion in latent HIV-1 infected J-Lat A2 and 11.1 T cell lines. We confirmed ADK, CHD9, CMSS1, EVI2B, EXOSC8, FAM19A, GRIK5, IRF2BP2, NF1, and USP15 as novel host factors involved in the maintenance of HIV-1 latency. Chromatin immunoprecipitation assays indicated that CHD9, a chromodomain helicase DNA-binding protein, maintains HIV-1 latency via direct association with the HIV-1 5′ long terminal repeat (LTR), and its depletion results in increased histone acetylation at the HIV-1 promoter, concomitant with HIV-1 latency reversal. FDA-approved inhibitors 5-iodotubercidin, trametinib, and topiramate, targeting ADK, NF1, and GRIK5, respectively, were characterized for their latency reversal potential. While 5-iodotubercidin exhibited significant cytotoxicity in both J-Lat and primary CD4^+^ T cells, trametinib reversed latency in J-Lat cells but not in latent HIV-1 infected primary CD4^+^ T cells. Importantly, topiramate reversed latency in cell line models, in latently infected primary CD4^+^ T cells, and crucially in CD4^+^ T cells from three people living with HIV-1 (PLWH) under suppressive antiretroviral therapy, without inducing T cell activation or significant toxicity. Thus, using an adaptation of a haploid forward genetic screen, we identified novel and druggable host factors contributing to HIV-1 latency.

## INTRODUCTION

Combination antiretroviral therapy (cART) has proven to effectively abrogate viral replication in HIV-1-infected patients and has substantially reduced AIDS-related mortality. However, cART is not curative and patients must remain on lifelong medication regimens, as interruption of the therapy leads to rapid rebound of viral replication (1). This is due to the persistence of a reservoir of latently infected cells, harboring replication-competent provirus blocked at the level of gene expression, which escape clearance by the immune system (2). Therapeutic strategies toward HIV-1 cures aim to inactivate, reduce, or completely eradicate the latent reservoir, such that, upon cessation of cART, the patient’s immune system can effectively control the infection or fully clear it (2). Strategies aiming to reduce or eliminate the reservoir rely on drugs, termed latency-reversing agents (LRAs), capable of inducing the latent HIV-1 infected cells to express viral genes to render infected cells susceptible to cytopathic effects and/or recognition and clearance by the immune system (3). Much focus has therefore been placed on finding small molecules for activating transcription of the latent HIV-1 provirus, which are not cytotoxic and do not induce harmful T cell activation or proliferation.

The identification of molecules capable of inducing HIV-1 gene expression has been largely accomplished using candidate approaches, which build on existing knowledge of transcription factors and cofactors that bind to and regulate transcription at the HIV-1 long terminal repeat (LTR) (4–6). One of the most clinically studied classes of molecules in HIV-1 latency reversal, histone deacetylase (HDAC) inhibitors (HDACis) such as SAHA (7), valproic acid (8), romidepsin (9), and M344 (10), targets HDACs, which have been shown to be recruited to the HIV-1 LTR by multiple transcription factors (11–13) to deacetylate histones and repress transcription. Similarly, agonists of the protein kinase C (PKC) pathway, such as prostratin and bryostatin, have been studied as activators of the HIV-1 LTR, as they induce nuclear localization and LTR binding of the NF-κB (p65/p50) heterodimer, a potent activator of HIV-1 transcription (14, 15). While this candidate approach has led to the identification of druggable targets or potential candidate LRAs, none of the LRAs currently under clinical investigation are capable of strong latency reversal in patients or lead to a reduction in the size of the latent HIV-1 reservoir (16, 17). Thus, to identify more potent and clinically relevant LRAs, it is critical to identify the full repertoire of functionally relevant host factors and pathways that play a role in the maintenance of latency.

Complementary to the approach of targeting candidate LTR-bound transcription factors and complexes for inhibition or activation, other studies have embarked on unbiased screens of small-molecule libraries to identify compounds capable of reversing HIV-1 latency (18). Alternatively, recent large-scale unbiased gene knockout/knockdown screens have been employed to unravel the molecular mechanisms of latent HIV-1 (19–28). RNA interference (RNAi) methods, which rely on the reduction of mRNA expression levels, have been widely used as screening platforms in mammalian cells (29). However, this method has been shown to suffer from serious limitations, including the presence of false positives due to off-target effects and the persistence of residual gene expression, which results in false negatives (30, 31). For example, several RNAi screens have been performed to identify host cell factors essential for HIV-1 infection and replication (19, 21, 22, 32); surprisingly, very little overlap was found between the lists of genes identified in these screens, pointing to the need for other screening methods that achieve complete inactivation of genes. Another large-scale unbiased gene disruption approach uses the precision of CRISPR-Cas9 targeting for complete gene knockouts (33). A recent screen using a lentiviral single guide RNA (sgRNA) sublibrary targeting nuclear proteins identified MINA53 as a possible latency-promoting gene (LPG) (28).

In mammalian cells, functional analysis via forward genetic screens and mutational analysis has largely been hampered due to diploidy, as, in somatic cells, when one copy of a gene essential for a cellular process is inactivated, the second copy often remains active and compensates for that partial loss. Pseudohaploid screens are based on KBM7, a chronic myelogenous leukemia (CML) cell line, which is haploid for all chromosomes except for chromosome 8 and a 30-Mb stretch of chromosome 15 (34), allowing for forward genetics in mammalian cells (35). Using gene-trap (GT) retrovirus-mediated mutagenesis for generating a library of gene knockouts in KBM7 cells enables unbiased loss-of-function screening in mutant cells for the identification of host genes essential to a specific cell function. Haploid screens have proven to be a powerful method to identify genes involved in drug import (36, 37), druggable target genes to treat cancer (38–40), key components of cellular pathways (41–45), and genes involved in the pathogenesis of various viruses (46–53) and susceptibility to toxins (35, 54, 55).

To identify novel host genes that could potentially serve as molecular targets for HIV-1 latency reversal, we performed insertional mutagenesis in latent HIV-1 infected KBM7 cells. Our screen identified 69 candidate genes, whose function is required for maintenance of HIV-1 latency but which are not essential to cell survival, a select subset of which we further validated using short hairpin RNA (shRNA) depletion in J-Lat T cell lines. We found candidate latency-promoting protein CHD9 to be enriched at the 5′ LTR in the latent state but to dissociate from the HIV-1 promoter after phorbol 12-myristate 13-acetate (PMA) treatment. We also found three genes in our candidate list, ADK, GRIK5, and NF1, to be targetable by existing FDA-approved small-molecule inhibitors, 5-iodotubercidin, trametinib, and topiramate, respectively, whose latency reversal potential we evaluated in J-Lat T cell lines A2 and 11.1 and in a primary cell model of HIV-1 latency. We found that the GRIK5 inhibitor topiramate reversed HIV-1 latency in both J-Lat T cell lines, primary CD4^+^ T cells harboring latent HIV-1, and CD4^+^ T cells from three people living with HIV-1 (PLWH) under suppressive antiretroviral therapy, without significant associated cytotoxicity or T cell activation, and thus presents an interesting potential novel LRA.

## RESULTS

### Establishment of a latent pseudohaploid cell line.

**FIG 1:**
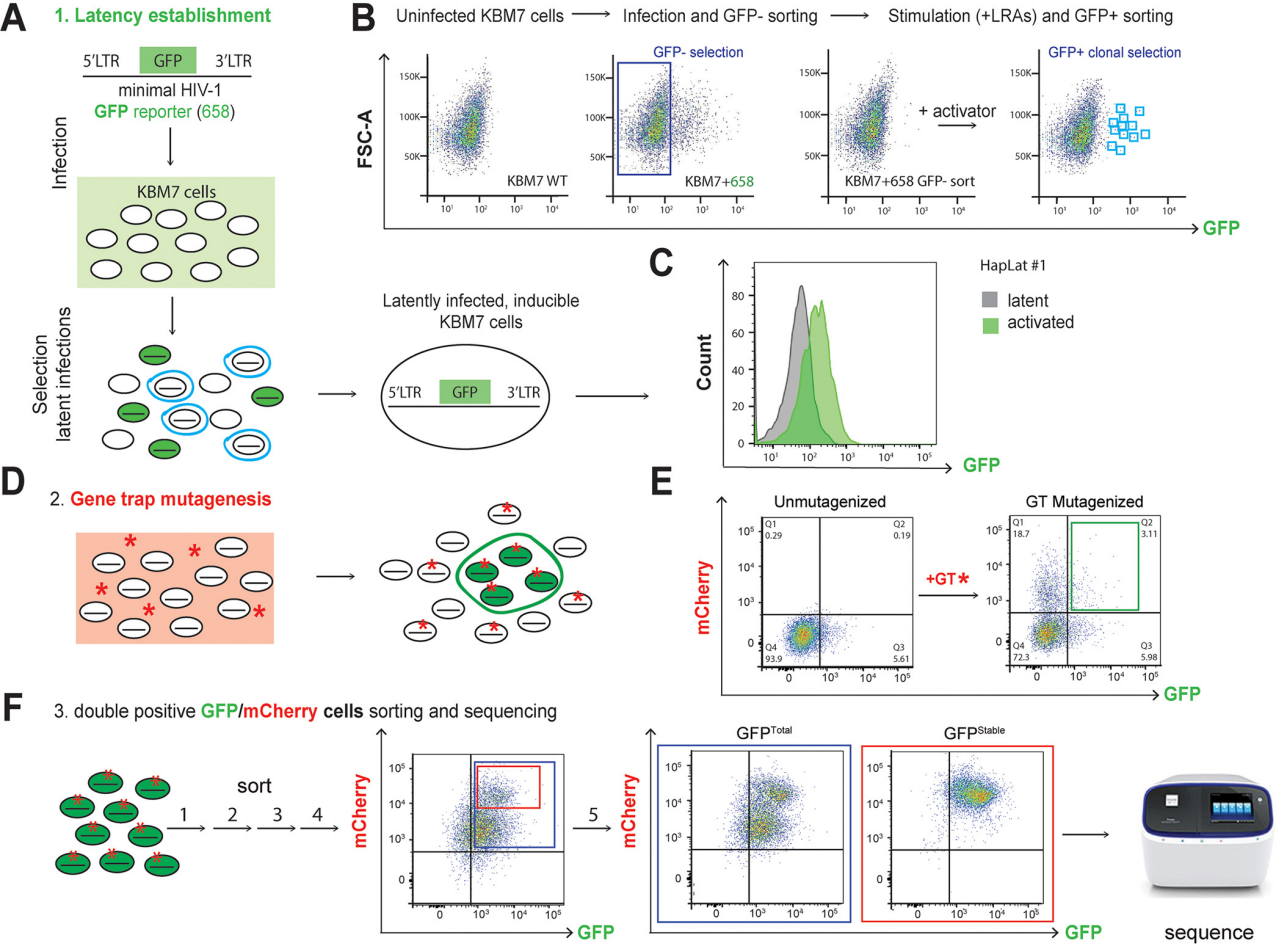
Schematic representation of the two-color haploid screening strategy for identification of novel host factors and cellular pathways involved in the maintenance of HIV-1 latency. (A) Scheme depicting the generation of a clonal latent haploid KBM7 cell line. To this end, haploid KBM7 cells were infected with a minimal HIV-1 virus harboring GFP (HIV-1 658). After stimulation, reactivated cells were sorted and left to expand and revert to a latent state. (B) FACS plots depicting the establishment of haploid latent HIV-1 infected KBM7 cell line (Hap-Lat). Parental KBM7 cells were infected with a minimal HIV-1 virus carrying a GFP reporter (HIV-1 658). GFP-negative cells, consisting of uninfected and latently infected cells, were sorted by FACS. The polyclonal cell pool was stimulated with a cocktail of latency reversal agents (LRAs), and reactivated cells were clonally sorted by FACS and expanded to generate haploid latent cell lines. WT, wild type. (C) Hap-Lat #1 displays low basal activity (GFP expression) but is effectively reactivated using LRAs. (D) Hap-Lat cells were mutagenized by infection with a gene-trap (GT) virus harboring an mCherry reporter. Cells infected with the GT reporter will be mCherry positive (red asterisk). Latently infected KBM7 cells that reactivate following GT mutagenesis will be double positive for GFP and mCherry (green cells, red asterisk). (E) Representative FACS plots demonstrating gating strategy for sorting double-positive cells (GFP, mCherry). (F) Double-positive cells (GFP, mCherry) are sorted in multiple rounds to eliminate cells stochastically reverting to a GFP-negative state. During these rounds of sorting, a stable and distinct double-positive subpopulation (GFP Sub) appears which was sorted separately.

To identify potentially druggable host genes as putative molecular targets for HIV-1 latency reversal, we generated pseudohaploid latent HIV-1 infected cells in which we performed insertional mutagenesis according to a strategy schematically depicted in Fig. 1A. A latent HIV-1 infected pseudohaploid KBM7 cell line was generated according to a previously described strategy used in Jurkat cells (56, 57) (Fig. 1B). Subsequently, haploid latent (Hap-Lat) cells harboring a latent integrated HIV-1-derived virus containing a green fluorescent protein (GFP) reporter were subjected to insertional mutagenesis using gene-trap virus carrying an mCherry reporter. Instead of using a lethality-selecting scheme, our system relies on fluorescence-activated cell sorting (FACS) to select for reactivated cells, marked by elevated GFP expression resulting from insertional mutagenesis of genes essential for maintenance of HIV-1 latency. Cells expressing both GFP and mCherry were FACS sorted and expanded for multiple rounds, after which GT insertion sites were mapped and identified by inverse PCR and high-throughput sequencing. To establish a latent HIV-1 infection in the pseudohaploid KBM7 system, near-haploid KBM7 cells were infected at a low multiplicity of infection (MOI) with the single-infectious-cycle HIV-1-derived virus LTR-GFP-LTR, in which GFP reporter expression is controlled by the activity of the HIV-1 promoter or 5′ LTR (Fig. 1A). After infection, the population of GFP-negative cells comprising mainly uninfected cells and putative latently infected cells were sorted by FACS and subsequently stimulated with 5 ng/μl tumor necrosis factor alpha (TNF-α) and 350 μM vorinostat. In response, a small percentage of existing latent HIV-1 infected cells were transcriptionally reactivated and expressed GFP. These GFP-positive cells were sorted by FACS as single cells and expanded (Fig. 1B). The resulting clonal latent KBM7 haploid lines were characterized by flow cytometry to determine GFP expression at basal and stimulated states. From the clonal lines established, Hap-Lat was selected for low basal activity and significant reactivation upon stimulation (Fig. 1C). To ensure maintenance of haploidy, Hap-Lat cells were periodically sorted to enrich for the 5% smallest cells (see Fig. S1A in the supplemental material). We further characterized reactivation kinetics of Hap-Lat cells in response to PMA under low and high serum concentrations (Fig. S1B and C) and investigated their reactivation potential in response to treatment with various classes of latency reversal agents in comparison with the more widely used J-Lat models (Fig. S1D and E). From this we concluded that the KBM7-derived pseudohaploid myeloid latent cell model Hap-Lat is an excellent model in which to perform a GT insertional mutagenesis screen.

10.1128/mBio.02980-21.1 FIG S1 Generation of haploid latent cell lines. (A) Ploidy as determined by propidium iodide staining of Hap-Lat #1 compared to Hap-Lat #1 cells cultured over a prolonged period and Jurkat cells. Haploidy of Hap-Lat cells can be maintained over time by periodic cell sorting of small cells. (B) Mean fluorescent intensity of GFP represented in absolute units in Hap-Lat wild-type (WT) cells cultured for 72 h in medium containing increasing fetal bovine serum percentages as indicated. (C) Mean fluorescent intensity of GFP represented in absolute units in Hap-Lat WT cells cultured for 72 h in medium containing increasing fetal bovine serum percentages as indicated and treated with PMA for 24 h. (D) Response of the Hap-Lat model to different classes of LRAs, including HDAC inhibitor, BAF inhibitor, BET inhibitor, and PKC agonist. (E) Response of the J-Lat A2 and 11.1 models to different classes of LRAs, including HDAC inhibitor, BAF inhibitor, BET inhibitor, and PKC agonist. (F) Infectivity by HIV-1 of KBM7 and other myeloid lineage cell lines, U937 and MV4, is poor compared to lymphocyte-derived cell lines (SupT1 and Jurkat) as shown by percent GFP-positive cells after 4 days of infections with minimal HIV-1 (658). (G) Nucleoside preincubation before and after supplementation during infection improves infectivity of KBM7 cells. (H) Schematic representing the mechanism of GT insertional mutagenesis. The inactive LTRs of MSCV flank a splice acceptor (SA) site, an mCherry reporter and a polyadenylation (pA) terminator. (I) After sorting and expansion, sorted double-positive cells tend to revert to a latent state as indicated by a loss of GFP signal. FACS plots obtained 1 day (left panel) and 5 days (right panel) after sorting. (J) Summary of the sorting timing intervals. Download FIG S1, TIF file, 1.0 MB. Copyright © 2021 Röling et al. 2021 Röling et al. https://creativecommons.org/licenses/by/4.0/ This content is distributed under the terms of the Creative Commons Attribution 4.0 International license.

### Gene-trap mutagenesis of Hap-Lat cells.

Hap-Lat cells were mutagenized by infection with a murine stem cell virus (MSCV)-derived viral gene-trap (GT) vector containing an inactivated 3′ LTR, an adenoviral splice-acceptor site, an mCherry reporter cassette, and a poly(A) terminator tail (35). Dendritic and myeloid cells are notoriously refractory to retroviral infection (58, 59). We indeed found that infectivity of KBM7 is poor compared to T-cell-derived cell lines SupT1 and Jurkat, as well as other myeloid-derived cell lines (Fig. S1F). SAMHD1, a nucleotide scavenger, has been identified as a causative restricting host factor that limits the free available pool of nucleotides for reverse transcription (60). To bypass SAMHD1-mediated restriction, we supplemented cells with 2 μM nucleosides (dNs), which increased GT infectivity by approximately 2-fold (Fig. S1G). GT preferentially inserts in the 5′ regions of genes (61), effectively knocking out gene expression by truncating the native transcript (Fig. S1H). For a full-scale mutagenesis experiment, approximately 200 million Hap-Lat #1 cells were mutagenized using two rounds of infection with GT-mCherry in the presence of exogenously supplied nucleosides. Infection of Hap-Lat with GT-mCherry effectively caused reactivation of a subpopulation of latent KBM7 cells (Fig. 1D and E). We reasoned that insertional mutagenesis in genes essential for maintenance of HIV-1 latency would result in Hap-Lat cells expressing the GFP reporter. We determined GT integrations in individual GFP/mCherry double-positive clones and estimated that the sequential infection resulted in 1 to 4 GT integrations per cell, with the majority of cells containing one integration as was reported before (35). Approximately 1 to 4% GFP/mCherry double-positive cells were subsequently sorted and expanded (Fig. 1E). During expansion, reactivated cells tend to revert to a latent state (Fig. S1I). To enrich for a more stable GFP-expressing, mCherry GT-containing double-positive cell population, cell sorting was repeated for multiple rounds (Fig. 1F and Fig. S1J). During the sequential rounds of sorting, we noted that a subpopulation within the total double-positive population displayed high levels of mCherry expression and remained stable in its GFP expression over culture time while other cells reverted to a GFP-negative state (Fig. 1F). To examine any potential biological differences between the two populations, we separately compared the stable GFP-positive, mCherry-high subpopulation, which we designated GFP^Stable^, enriched after a final (fifth) round of sorting (Fig. S1J), to the total double-positive population, which was designated GFP^Total^ (Fig. 1F). Genomic DNA extracted from GFP^Total^ and GFP^Stable^ obtained in the fifth round of sorting was used to determine GT integrations, while that of a pool of unsorted GT-infected cells was used as a reference.

### Mapping of insertion sites to identify host factors maintaining HIV-1 latency.

**FIG 2:**
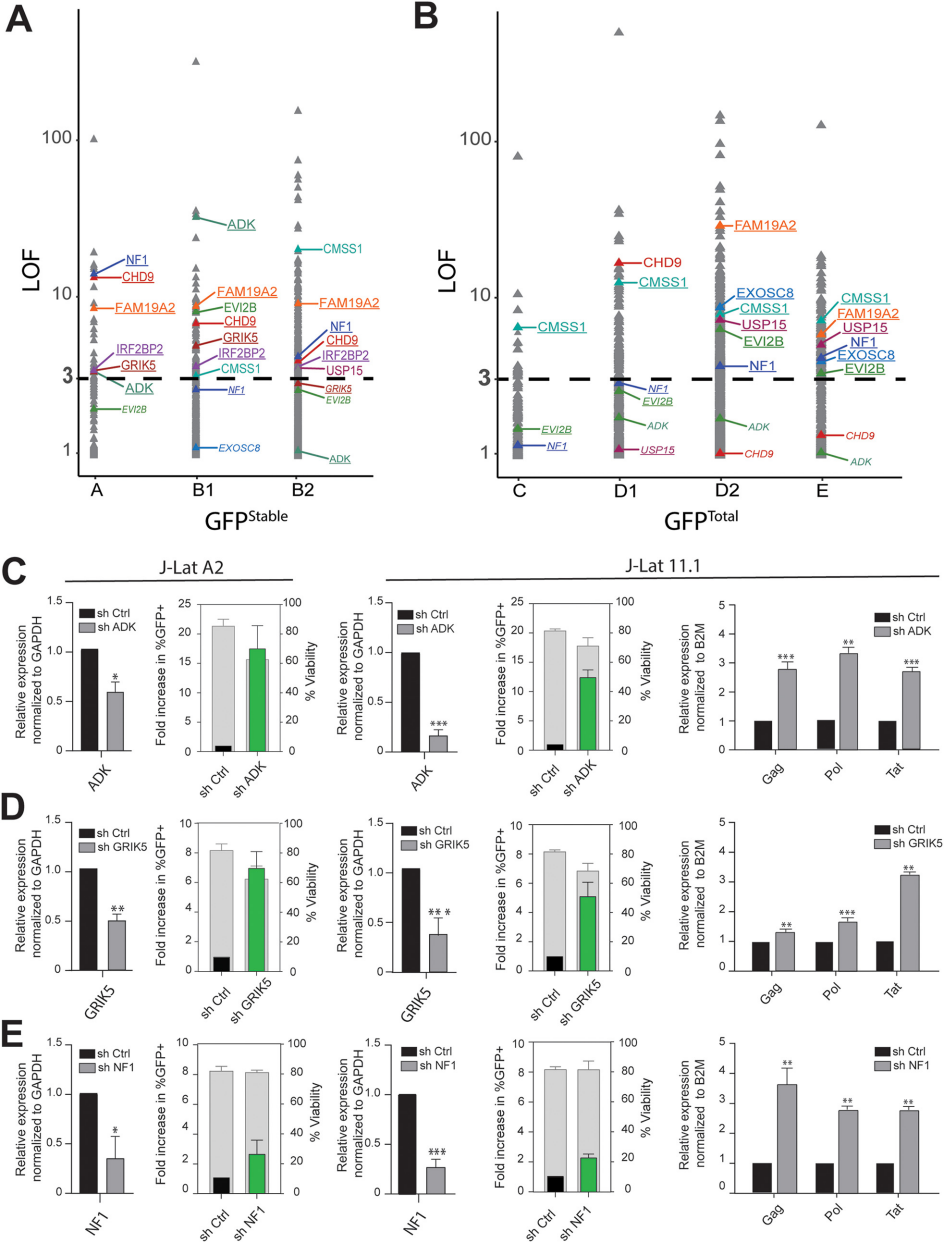
Identification and validation of candidate host factors. (A) LOF scores of genes in the GFP^Stable^ population (samples A, B1, and B2); validated candidates are indicated. Genes with LOF scores of >3 are in large font, and underlined genes comply with our candidate gene selection criteria and have a LOF score of >3 in at least two biological replicates within either the GFP^Stable^ or GFP^Total^ population, while genes with a LOF score of <3 but complying with our selection criteria based on other samples are depicted in small font and italics. (B) LOF scores of genes in the GFP^Total^ population (samples C, D1, D2, and E); markings are as in panel A. (C to E) Functional validation of candidate hits ADK (C), GRIK5 (D), and NF1 (E) by shRNA-mediated depletion followed by determination of latency reversal by flow cytometry and RT-PCR in latently infected J-Lat A2 (left panels) and 11.1 (right panels) cells. Flow cytometry bar plots: green bars show the percentage of GFP-positive cells after knockdown over control (black bar), left *y* axis, whereas gray bars show cell viability, right *y* axis. Viral reactivation is confirmed by RT-qPCR for viral genes Tat, Gag, and Pol in J-Lat 11.1 cells. Statistical significance was calculated using ratio-paired *t* test and multiple-comparison *t* test on log_2_-transformed fold changes: *, *P* < 0.05; **, *P* < 0.01; ***, *P* < 0.001.

To determine the host sequences flanking the GT insertion sites, inverse PCR with primers annealing to internal sequences in the gene-trap vector followed by amplification was performed. The amplified products were processed for high-throughput sequencing (Fig. 1F). For GFP^Stable^ two biological replicates, samples A and B, were generated. For GFP^Total^ three biological replicates were generated, samples C, D, and E. To estimate the sampling depth of our GT, we resequenced GFP^Stable^ sample B and GFP^Total^ sample D at greater depth. The resulting next-generation sequencing (NGS) data sets were processed for candidate gene identification. A previously described method to analyze GT data, HaSAPPy (Haploid Screen Analysis Package in Python), was rigorously reimplemented, appended with additional steps for quality control, library normalization, and optimized resolution for the selection of integration sites (62). HaSAPPY assigns a Local Outlier Factor (LOF) score to each gene in a sample based on a triplet score derived from the number of putative GT integrations in the sample compared to the reference. For each population, we compiled all genes with an LOF score of >3 from each replicate and obtained 686 hits for GFP^Total^ and 382 hits for GFP^Stable^. One hundred eighty-three genes were common to the two populations (Fig. S2A). Next, we investigated any potential biological basis for the difference between GFP^Total^ and GFP^Stable^. Since expression levels of the integrated mCherry reporter appear to be on average higher in the GFP^Stable^ population than in the GFP^Total^ population, we wondered if expression levels of the targeted genes were higher in GFP^Stable^. We obtained recently published KBM7 gene expression data (63) and found no substantial difference in the average level of expression between the two populations (Fig. S2B). To determine if there was any difference in the functionality of the GT target genes found in GFP^Total^ and GFP^Stable^, we performed enrichment analysis using Gene Ontology (GO) terms and found no substantial differences in enrichment for biological process and molecular function ontologies (Fig. S2C and D). Similarly, the two populations contain a comparable fraction (32.7%) of integrations within noncoding or antisense genes (Fig. S2E). Finally, we cross-referenced the GT target genes found in GFP^Total^ and GFP^Stable^ populations to the HIV-1 interaction database (https://www.ncbi.nlm.nih.gov/genome/viruses/retroviruses/hiv-1/interactions/) and found that the GFP^Total^ and the GFP^Stable^ populations contain similar fractions (21.9% and 20.2%, respectively) of genes previously reported to be involved in HIV-1 biology, fractions which are substantially higher than the 7.4% found for the complete list of ENSEMBL genes (Fig. S2F). In order to limit our extensive list of candidate genes for follow-up functional validation, we applied more stringent thresholds for each population and defined candidate genes as having an LOF score equal to or greater than 3 in at least 2 biological replicate samples. We thus identified 19 candidate genes in the GFP^Stable^ population and 55 in the GFP^Total^ population (Fig. 2A and B and Table 1).

**TABLE 1:**
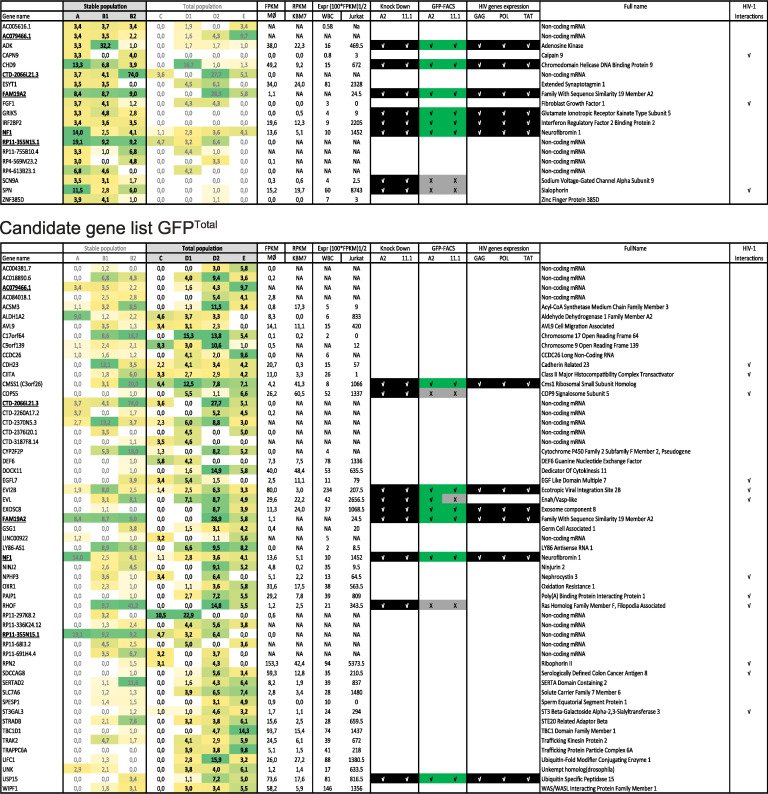
Prioritized candidate list for the GFP^Total^ and the GFP^Stable^ populations^*a*^

a Prioritized candidate genes are listed in column 1. Genes found in both the GFP^Total^ and the GFP^Stable^ populations are underlined in both lists. LOF scores are indicated in columns 2 (GFP^Stable^ population) and 3 (GFP^Total^ population). Color coding indicates LOF score (light yellow, low LOF score, to green, high LOF score). Boldface indicates an LOF score equal to or greater than 3. Prioritized candidate genes are defined as having a LOF score equal to or greater than 3 in at least 2 independent samples. Expression in macrophages (Mϕ) (GSE93155), KBM7 cells (GSE47428), leukocytes (WBC) (https://gtexportal.org/home/), and Jurkat cells (64) is shown in columns 4 and 5; NA, not available in data set. Results of shRNA-mediated knockdown validation experiments of selected candidate genes in A2 and 11.1 J-Lat cell models are indicated in columns 6 to 8 (**√** in black background indicates successful knockdown or successful induction of HIV-1 GAG, POL, or TAT mRNA expression, **√** in green background indicates induction of GFP expression, X in gray background indicates no induced GFP expression). Previously reported interactions with HIV-1 according to the HIV-1 interaction database are indicated in column 10 by a **√** (https://www.ncbi.nlm.nih.gov/genome/viruses/retroviruses/hiv-1/interactions/). Additional abbreviations: FPKM, fragments per kilobase per million; RPKM, reads per kilobase per million; CoA, coenzyme A.

10.1128/mBio.02980-21.2 FIG S2 Characterization of gene-trap integration candidate genes. (A) Venn diagram depicting all genes with an LOF score of >3 in one replicate from the GFP^Total^ (686 hits) and the GFP^Stable^ (382 hits) populations. One hundred eighty-three genes are common to both populations. (B) Expression levels of genes in the GFP^Total^ and the GFP^Stable^ populations. No significant differences in the average expression levels of genes between the two populations are found (Mann-Whitney test). (C) GO-slim term analysis for biological process of genes in the GFP^Total^ and the GFP^Stable^ populations. No substantial differences in GO-slim term enrichment are found. (D) GO-slim term analysis for molecular function of genes from both populations. (E) Percentages of noncoding genes present among genes in the GFP^Total^ and the GFP^Stable^ populations are similar. (F) Percentages of genes present in the HIV-1 interaction database (https://www.ncbi.nlm.nih.gov/genome/viruses/retroviruses/hiv-1/interactions/) among genes in the GFP^Total^ and the GFP^Stable^ populations are similar but higher than the total ENSEMBL gene list. Download FIG S2, TIF file, 2.7 MB. Copyright © 2021 Röling et al. 2021 Röling et al. https://creativecommons.org/licenses/by/4.0/ This content is distributed under the terms of the Creative Commons Attribution 4.0 International license.

### Candidate list validation.

**FIG 3:**
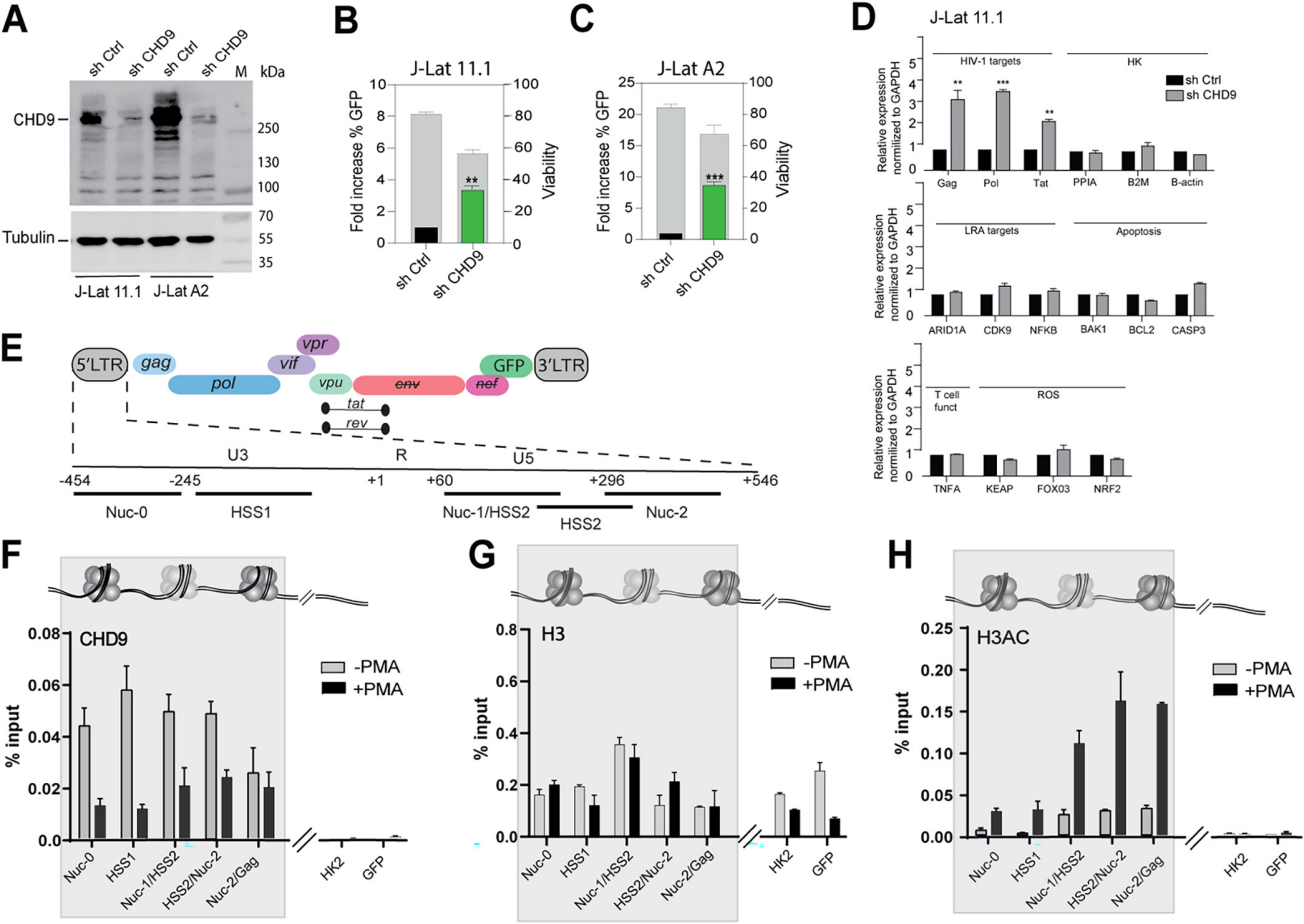
CHD9 regulates HIV-1 latency in J-Lat A2 and J-Lat 11.1 cells. (A) Western blot assay for CHD9 in control and CHD9 shRNA-depleted J-Lat 11.1 and A2 cells, with α-tubulin as a loading control. (B) Flow cytometry bar plots demonstrating latency reversal after CHD9 shRNA depletion in J-Lat 11.1 cells: green bars indicate the percentage of GFP-positive cells after knockdown over control (black bar), left *y* axis, whereas gray bars show cell viability, right *y* axis. (C) Latency reversal after CHD9 shRNA depletion in J-Lat A2 cells. (D) RT-qPCR data from J-Lat 11.1 cells transduced with CHD9 shRNA for the HIV-1 viral genes Tat, Gag, and Pol; housekeeping genes PPIA, B-ACT, and B2M; LRA targets ARID1A, CDK9, and NFKB; apoptosis genes BAK1, BCL2, and CASP3; T cell functionality gene TNF-α; and reactive oxygen species (ROS) genes KEAP, FOXO3, and NRF2. Data are normalized to GAPDH and represented as fold increase over sh Control. Statistical significance was calculated using ratio-paired *t* test and multiple-comparison *t* test on log_2_-transformed fold changes: *, *P* < 0.05; **, *P* < 0.01; ***, *P* < 0.001. (E) Schematic of HIV-1 genome. 5′ LTR region further segmented into the U3, R, and U5 regions. Amplicons used in ChIP-qPCR experiments are indicated. (F) ChIP-qPCR analysis of CHD9 binding to the HIV-1 5′ LTR in untreated and PMA-stimulated J-Lat 11.1 cells. Data are represented as percentage of the input. (G) ChIP-qPCR analysis of histone H3 occupancy at the HIV-1 5′ LTR in untreated and PMA-stimulated J-Lat 11.1 cells. Data are represented as percentage of the input. (H) ChIP-qPCR analysis of histone H3 acetylation at the HIV-1 5′ LTR in untreated and PMA-stimulated J-Lat 11.1 cells. (E to H) Data represent the average (±SD) from two technical replicates.

Since our bioinformatics analysis did not reveal a defining difference between the GFP^Total^ and the GFP^Stable^ populations, we decided to proceed with functional assays using shRNA-mediated depletion of candidate genes obtained from both populations. To prioritize the candidate genes found in the KBM7 haploid screen for functional validation in the more biologically relevant Jurkat T-cell-based HIV-1 latency models J-Lat A2 and 11.1, we focused on protein-coding genes and took into account LOF scores as well as gene expression in white blood cells (as extracted from the GTEx portal [https://gtexportal.org/home/] or Illumina’s Human BodyMap 2.0 project [http://www.ensembl.info/2011/05/24/human-bodymap-2-0-data-from-illumina/]) and the Jurkat T cell model (64) (Table 1). Eight genes from GFP^Stable^ and nine genes from GFP^Total^ were selected as candidates for validation using shRNA-mediated depletion in J-Lat A2 and J-Lat 11.1 cells. These cells contain a latent HIV-1-derived GFP reporter driven by the 5′ HIV-1 LTR and are well-established model systems for HIV-1 latency (56, 65). While J-Lat A2 cells contain an integrated latent LTR-Tat-IRES (internal ribosome entry site)-GFP virus, J-Lat 11.1 cells contain an envelope-defective full-length HIV-1 genome expressing GFP in place of Nef. We used flow cytometry to assess reactivation as measured by GFP expression and extracted RNA to assess knockdown of the targeted candidate genes and expression of the HIV-1 genes GAG, POL, and TAT by reverse transcription-quantitative PCR (RT-qPCR) (Fig. 2C to E and Fig. S3 and S4). Significant latency reversal upon candidate gene knockdown was observed for 10 out of 15 genes depleted by shRNA in both J-Lat A2 and J-Lat 11.1 cells (ADK, GRIK5, and NF1 [shown in Fig. 2C to E], CHD9 [Fig. 3A to C], and CMSS1, EVI2B, EXOSC8, FAM19A, IRF2BP2, and USP15 [Fig. S3 and Table 1]). Knockdown of SCN9A, RHOF, SPN, COPS5, and EVL did not result in significant latency reversal in one or both J-Lat models (Fig. S4). We also validated latency reversal in J-Lat 11.1 cells depleted of ADK, NF1, GRIK5, and CHD9 at the protein level by Western blotting of Gag (Fig. S5A to C). These results demonstrate that a significant proportion of the candidate genes found in our myeloid-derived KBM7 haploid screen play a role in maintenance of HIV-1 latency in the more relevant T-cell-derived J-Lat A2 and J-Lat 11.1 HIV-1 latency models.

10.1128/mBio.02980-21.3 FIG S3 Functional validation of positive candidates. Reactivation of HIV-1 in J-Lat A2 and 11.1 cell lines was assessed by measuring the percentage of cells expressing GFP (green bars) and cell viability (gray bars) using flow cytometry. Efficacy of knockdown by shRNA was quantitated in J-Lat 11.1 cells by RT-qPCR (left panel) as well as expression of viral genes Tat, Pol, and Gag. RT-qPCR data (right panel) are presented as mean ± SD normalized to the control. Statistical significance was calculated using ratio-paired *t* test and multiple-comparison *t* test on log_2_-transformed fold changes (*, *P* < 0.05, **, *P* < 0.01; ***, *P* < 0.001). Download FIG S3, TIF file, 1.3 MB. Copyright © 2021 Röling et al. 2021 Röling et al. https://creativecommons.org/licenses/by/4.0/ This content is distributed under the terms of the Creative Commons Attribution 4.0 International license.

10.1128/mBio.02980-21.4 FIG S4 Functional validation of false-positive candidates. Efficacy of knockdown by shRNA was quantitated by RT-qPCR. Reactivation of HIV-1 was assessed by measuring the percentage of cells expressing GFP (green bars) and cell viability (gray bars). RT-qPCR data are presented as mean ± SD normalized to the control. Statistical significance was calculated using ratio-paired *t* test and multiple-comparison *t* test on log_2_-transformed fold changes (*, *P* < 0.05; **, *P* < 0.01; ***, *P* < 0.001). Download FIG S4, TIF file, 2.0 MB. Copyright © 2021 Röling et al. 2021 Röling et al. https://creativecommons.org/licenses/by/4.0/ This content is distributed under the terms of the Creative Commons Attribution 4.0 International license.

10.1128/mBio.02980-21.5 FIG S5 Additional data on the functional characterization of CHD9. (A) Western blot assay for Gag p24 in control, CHD9, and NF1 shRNA-depleted J-Lat 11.1 cells. Tubulin was used as a loading control. (B) Western blot assay for Gag p24 in control, ADK, and CHD9 shRNA-depleted J-Lat 11.1 cells. Tubulin was used as a loading control. (C) Western blot assay for Gag p24 in control and GRK5 shRNA-depleted J-Lat 11.1 cells. Tubulin was used as a loading control. (D) Efficacy of CHD9 knockdown in J-Lat A2 cells and J-Lat 11.1 cells by shRNA was quantitated by RT-qPCR. (E) Biological replicate of a ChIP-qPCR analysis of CHD9 binding to the HIV-1 5′ LTR in untreated J-Lat 11.1 cells and PMA-stimulated cells (similar to data from Fig. 3G and H). Data are represented as percentage of the input and represent the average (±SD) from two technical replicates. Download FIG S5, TIF file, 0.6 MB. Copyright © 2021 Röling et al. 2021 Röling et al. https://creativecommons.org/licenses/by/4.0/ This content is distributed under the terms of the Creative Commons Attribution 4.0 International license.

### CHD9 is an LTR-associated repressor of HIV-1 transcription.

Interestingly, two genes, CIITA and CHD9, from our candidate list are associated with the GO term DNA binding (GO:0003677). CIITA is a well-established factor involved in HIV-1 expression and has been previously shown to inhibit Tat function and hence viral replication (66, 67). The chromodomain helicase DNA binding protein 9 (CHD9) is a member of an ATP-dependent chromatin remodeler family, the members of which modulate DNA-histone interactions and positioning of nucleosomes and play key roles in stem cell regulation, development, and disease (68). Previously, we have shown that chromatin remodeling by another ATP-dependent remodeler, the BAF complex, plays a crucial role in maintenance of HIV-1 latency and its reactivation (57). We therefore decided to further characterize the role of CHD9 in regulating HIV-1 gene expression. We knocked down CHD9 using a lentivirally transduced shRNA and verified its depletion in both J-Lat A2 and J-Lat 11.1 cells at the protein level by Western blotting (Fig. 3A) and RT-qPCR (Fig. S5D). Depletion of CHD9 led to a significant reversal of latency, as shown by an increase in the percentages of GFP-positive cells (Fig. 3B and C). Latency reversal was also confirmed by increased expression of viral genes Gag, Pol, and Tat at the RNA level in J-Lat 11.1 while expression of the housekeeping genes PPIA, B-ACT, and B2M remained unaltered (Fig. 3D). We also investigated whether depletion of CHD9 would alter gene expression in a pleiotropic manner. CHD9 knockdown did not lead to changes in expression of LRA target genes such as BAF inhibitors (ARID1A), elongation inducers (CDK9), and NF-κB activators (NFKB); genes involved in apoptosis (BCL2, BAK1, and CASP3); T cell functionality (TNFA); or reactive oxygen species (ROS) (FOXO3, KEAP, and NRF2) (Fig. 3D). Overall, we observed that CHD9 knockdown does not lead to production, T cell functionality, and latency reversal upon treatment with other LRAs (Fig. 3D).

To characterize a potential direct association of CHD9 with the latent HIV-1 5′ LTR, we performed chromatin immunoprecipitation (ChIP) in latent and PMA-activated 11.1 J-Lat cells. The positions of nucleosomes within the latent HIV-1 LTR are rigid, with positioned nucleosomes Nuc-0, Nuc-1, and Nuc-2 separated by DNase I-sensitive regions HSS1 and HSS2, respectively, visually summarized in Fig. 3E. CHD9 was found to be enriched throughout the HIV-1 LTR in latent 11.1 J-Lat cells, and this association was significantly decreased after LTR activation by PMA treatment (Fig. 3F and Fig. S5E). No significant enrichment was observed at the HIV-1 internal control region (the GFP reporter) or the unrelated gene locus (HK2) (Fig. 3F and Fig. S5E). As expected, PMA treatment led to an increase in histone H3 acetylation, a mark of active chromatin, over the HIV-1 5′ LTR (Fig. 3G and H and Fig. S5E). Our data demonstrate a role for CHD9 in the repression of HIV-1 gene expression and maintenance of latency via direct recruitment to and association with the HIV-1 5′ LTR.

### Pharmacological targeting of ADK, GRIK5, and NF1.

With the aim of identifying potential latency-reversing agents (LRAs), we performed a literature search and identified three candidate genes, ADK, GRIK5, and NF1, all present in the GFP^Stable^ list, for which FDA-approved small-molecule inhibitors are available. We therefore examined the latency reversal potential of the ADK inhibitor 5-iodotubercidin, the GRIK5 inhibitor topiramate, and the NF1 inhibitor trametinib. Adenosine kinase (ADK) is a phosphotransferase that converts adenosine into 5′-AMP and thus plays a major role in regulating the intracellular and extracellular concentrations of adenosine, activation of specific signaling pathways, and bioenergetic and epigenetic functions (69, 70). 5-Iodotubercidin is a purine derivative that inhibits ADK by competing with adenosine for binding to the enzyme (71). Glutamate ionotropic receptor kainate type subunit 5 (GRIK5) is a subunit of the tetrameric kainate receptor (KAR), a subgroup of ionotropic glutamate receptors. GRIK5, together with GRIK4, binds glutamate, whereas subunits GRIK1 to -3 form functional ion channels (72). Topiramate is an FDA-approved GRIK5 inhibitor employed as an antiepileptic drug and is used to manage seizures and prevent migraines (73). Neurofibromin 1 (NF1) is ubiquitously expressed; however, its highest levels are found in cells of the central nervous system, and it has been described to function as a negative regulator of the Ras signal transduction pathway (74, 75). Trametinib is an NF1 inhibitor which is also known as a mitogen-activated protein kinase (MAPK) kinase (MEK) inhibitor with anticancer activity and is FDA approved for use in metastatic malignant melanoma (76).

**FIG 4:**
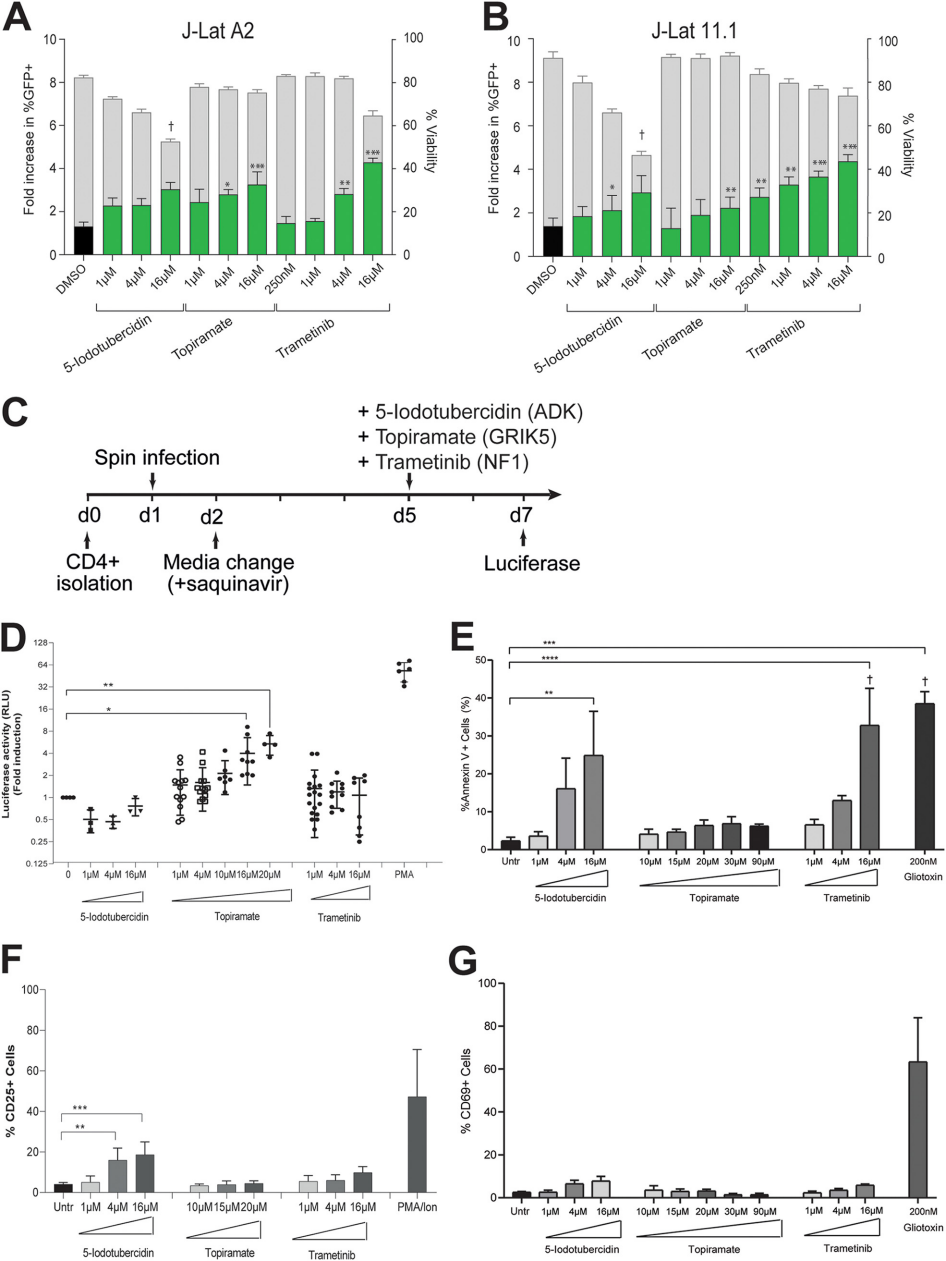
HIV-1 latency reversal by small-molecule inhibitors of three candidate genes, ADK, GRIK5, and NF1. (A) Latency reversal potential upon 48-h treatment of J-Lat A2 cells with increasing concentrations of 5-iodotubercidin (ADK inhibitor), topiramate (GRIK5 inhibitor), and trametinib (NF1 inhibitor) was evaluated by flow cytometry. Treatment with DMSO (black bar) is used as a negative control. Percentage of GFP-positive cells is indicated by green bars (left *y* axes), and cell viability is indicated by gray bars (right *y* axis). (B) Latency reversal potential upon 48-h treatment of J-Lat 11.1 cells. (C) Schematic representation of candidate LRA treatment in a primary cell model of latent HIV-1 infection. CD4^+^ T cells are isolated on day 0 and spin infected on day 1. On day 2 virus is removed by medium change in the presence of saquinavir. Latently infected cells are stimulated with candidate LRAs on day 5, and HIV-1 reactivation is evaluated at day 7. (D) Latency reversal as measured by luciferase activity in a primary cell model of HIV-1 latency after treatment with 5-iodotubercidin, trametinib, and topiramate in different concentrations. Plots show the fold increase in luciferase activity, measured in relative light units (RLU), after treatment with different concentrations of 5-iodotubercidin (ADK inhibitor), topiramate (GRIK5 inhibitor), and trametinib (NF1 inhibitor). Each dot represents a single measurement, and black horizontal lines show the average fold increase for each treatment. Averaged data from at least 3 independent experiments performed using each time two different donors (totaling at least 6 different donors). PMA was used as a positive control. Statistical significance was calculated using *t* test: *, *P* < 0.05; **, *P* < 0.005; ***, *P* < 0.0005. (E) Percentage of cells expressing apoptosis marker annexin V in primary CD4^+^ T cells upon treatment with candidate LRAs for 48 h. Treatment with a toxic concentration of gliotoxin (GTX), 200 nM, was used as a positive control. Experiments were performed in uninfected cells obtained from 6 healthy donors. Data are presented as mean ± SD from three independent experiments. The † symbol indicates low viability. (F) Percentage of cells expressing marker of cell activation CD25 in primary CD4^+^ T cells from 6 healthy donors; data are presented as mean ± SD from three independent experiments of 2 different healthy donors upon treatment with candidate LRAs for 48 h. Treatment with PMA/ionomycin is used as a positive control. (G) Percentage of cells expressing marker of cell activation CD69 in primary CD4^+^ T cells from 6 healthy donors; data are presented as mean ± SD from three independent experiments of 2 different healthy donors upon treatment with candidate LRAs for 48 h. Treatment with PMA/ionomycin is used as a positive control. Statistical significance was calculated using one-way ANOVA, multiple-comparison test. Asterisks indicate the level of significance. (**, *P* < 0.01; ***, *P* < 0.001).

We examined the effects of treatment with 5-iodotubercidin, topiramate, and trametinib in J-Lat 11.1 and J-Lat A2 cells, and a primary CD4^+^ T cell model of latency, on latency reversal. Treatment with the ADK inhibitor 5-iodotubercidin resulted in only a moderate induction of latency reversal in both A2 and 11.1 J-Lat cells and was accompanied by significant toxicity, especially at high concentrations (16 μM) (Fig. 4A and B). Only at a 5-iodotubercidin concentration of 4 μM in 11.1 J-Lat cells was a significant increase in GFP-positive cells with acceptable viability observed (Fig. 4A and B). Treatment of J-Lat A2 and 11.1 cells with the GRIK5 inhibitor topiramate or the NF1 inhibitor trametinib resulted in significant, concentration-dependent latency reversal (Fig. 4A and B). Importantly, neither topiramate nor trametinib displayed significant toxicity in comparison with the dimethyl sulfoxide (DMSO) vehicle control group, as measured by gating for live cells. In particular, treatment of J-Lat 11.1 cells with trametinib resulted in significant latency reversal at all evaluated concentrations (250 nM, 1 μM, 4 μM, and 16 μM), with minimal associated toxicity (Fig. 4B).

Next, we evaluated the latency reversal potential of the inhibitors in a more clinically relevant primary *ex vivo* infection latency model, in which CD4^+^ T cells are infected with a full-length non-replication-competent HIV-1 virus driving expression of a luciferase reporter (Fig. 4C) (77). After spin infection, cells are allowed to rest for 4 days and are treated with the different compounds, as indicated, for 48 h, followed by measurement of HIV-1 LTR-driven luciferase activity and staining for annexin-positive cells and T cell activation markers CD25 and CD69 (Fig. 4D to G and Fig. S6). Similar to what was observed for the A2 and 11.1 J-Lat cell lines, treatment of HIV-1-infected latent primary CD4^+^ T cells with the ADK inhibitor 5-iodotubercidin did not result in significant latency reversal (Fig. 4D), while it produced significant toxicity at the highest concentration used, as indicated by annexin V staining (Fig. 4E). Treatment with the GRIK5 inhibitor topiramate, at a concentration of 16 and 20 μM, resulted in significant (*P* < 0.05) reversal of latency, as measured by a 3- to 6-fold increase in mean luciferase activity compared to untreated controls (Fig. 4D). Topiramate treatment did not significantly affect CD4^+^ T cell viability after 48 h, while control treatment with a toxic (200 nM) concentration of gliotoxin (GTX) caused apoptosis of primary CD4^+^ T cells, as evidenced by an increase in the percentage of annexin V-positive cells, as was observed previously (Fig. 4E) (78). In contrast to what was observed in the A2 and 11.1 J-Lat cell lines, treatment of latently infected CD4^+^ T cells with the NF1 inhibitor trametinib did not lead to a significant reversal of latency (Fig. 4D), while viability was only moderately affected (Fig. 4E).

10.1128/mBio.02980-21.6 FIG S6 Characterization of T cell activation by candidate LRAs. (A) FACS plot of staining for T cell activation marker CD69 after treatment of primary CD4^+^ T cells from 6 healthy donors with candidate LRAs (5-iodotubercidin, topiramate, and trametinib). Treatment with PMA/ionomycin is used as a positive control (plots correspond to data from Fig. 4F). (B) FACS plot of staining for T cell activation marker CD25 after treatment with candidate LRAs (plots correspond to data from Fig. 4G). Download FIG S6, TIF file, 1.9 MB. Copyright © 2021 Röling et al. 2021 Röling et al. https://creativecommons.org/licenses/by/4.0/ This content is distributed under the terms of the Creative Commons Attribution 4.0 International license.

For LRA candidates to be viable in a clinical setting, it is crucial that they reactivate latent HIV-1 without inducing activation of CD4^+^ T cells. Therefore, we examined the potential of our candidate LRAs to induce expression of the activation markers CD25 and CD69 in treated uninfected CD4^+^ T cells from healthy individuals. While the ADK inhibitor 5-iodotubercidin resulted in markedly elevated expression of the activation markers, the GRIK5 inhibitor topiramate and NF1 inhibitor trametinib did not induce significant induction of the T cell activation markers CD25 and CD69 (Fig. 4F and G and Fig. S6A and B). As expected, PMA/ionomycin treatment substantially activated T cells (Fig. 4F and G and Fig. S6A and B). Finally, to determine if GRIK5 inhibition is also able to reverse latency in the myeloid lineage, we revisited our KBM7-derived Hap-Lat line and performed sh-mediated GRIK5 knockdown (Fig. S6C) and GRIK5 inhibition using topiramate (Fig. S6D) and demonstrated latency reversal with limited loss of viability in myeloid cells upon GRIK5 depletion or inhibition. Taken together, our data indicate that the FDA-approved GRIK5 inhibitor topiramate reverses HIV-1 latency in a variety of T cell models of latency, without induction of T cell activation and with limited cytotoxicity, and therefore justifies further investigation in CD4^+^ T cells from PLWH under suppressive antiretroviral therapy.

### The FDA-approved compound topiramate reverses latency in CD4^+^ T cells from PLWH.

**FIG 5:**
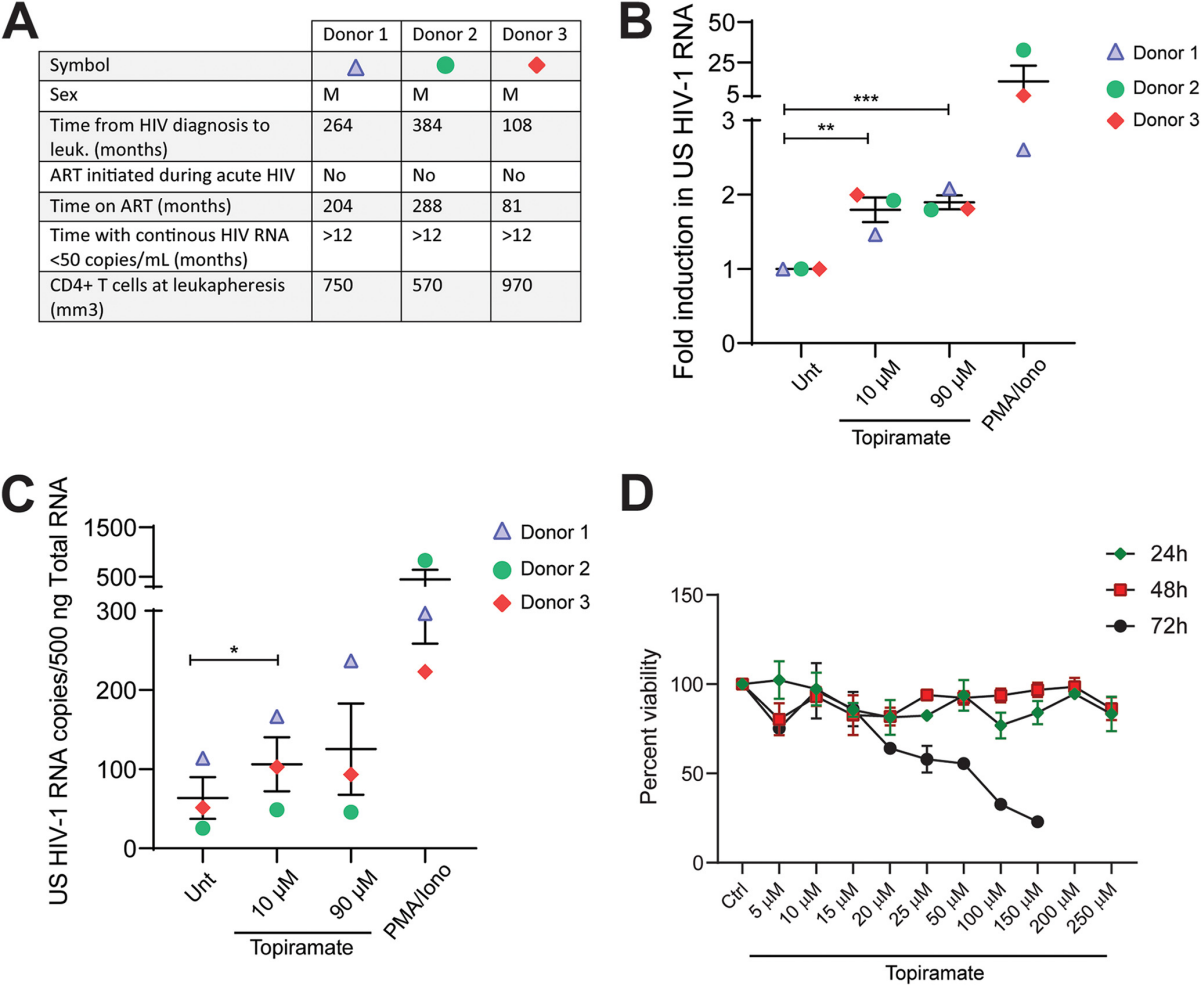
The FDA-approved drug topiramate reverses HIV-1 latency *ex vivo* in cells obtained from cART-suppressed PLWH with limited toxicity. (A) Clinical characteristics corresponding to the aviremic HIV-1-infected study participants described in panels B and C. (B) Fold change in cell-associated unspliced HIV-1 RNA in CD4^+^ T cells isolated from PBMCs from aviremic HIV-1-infected donors after treatment with different concentrations of topiramate for 24 h. PMA/ionomycin was used as a positive control. Statistical significance was calculated using two-tailed *t* test: *, *P* < 0.05; **, *P* < 0.01; ***, *P* < 0.001. (C) Induction of unspliced (US) HIV-1 RNA copies per 500 ng total RNA in CD4^+^ T cells from HIV-1-infected individuals as presented in panel B. PMA/ionomycin was used as a positive control. Statistical significance was calculated using two-tailed *t* test: *, *P* < 0.05. (D) Viability assay in primary CD4^+^ T cells obtained from healthy donors after treatment with topiramate as indicated for 24, 48, and 72 h. CD4^+^ T cells were isolated from healthy individuals and treated as indicated for 24, 48, and 72 h. Viability was assessed by alamarBlue viability staining. Treatment readings are normalized to untreated control, represented as 100%.

As shown by our data in *ex vivo* models of HIV-1 latency, topiramate is a potentially interesting candidate in the context of HIV-1 latency reversal. However, *ex vivo* models of latency do not fully represent the breadth and complexity of HIV-1 viral reservoirs *in vivo*, and other model systems are required to better assess the potential effect of novel LRAs. In order to further characterize the role of the FDA-approved drug topiramate against HIV-1 in a more relevant system, we assessed its efficacy in reversing HIV-1 latency in *ex vivo*-treated primary CD4^+^ T cells obtained from three aviremic HIV-1-infected donors (Fig. 5A). CD4^+^ T cells from PLWH were treated with different concentrations of topiramate for 24 h, and latency reversal was analyzed by determining cell-associated (CA) HIV-1 RNA using nested qPCR targeting the unspliced (US) HIV-1 RNA. Our data show that topiramate is able to significantly reactivate HIV-1 latency in *ex vivo*-treated CD4^+^ T cells from PLWH as shown by a significant change in fold induction and increase in total US HIV-1 RNA (Fig. 5B and C). In our system, topiramate was able to induce up to a 2-fold increase in CA HIV-1 US RNA at 24 h after treatment (Fig. 5B) and correlated with an average of up to 42% of the activation observed with PMA/ionomycin (Fig. S7).

10.1128/mBio.02980-21.7 FIG S7 The FDA-approved drug topiramate reverses HIV-1 latency *ex vivo* in cells obtained from cART-suppressed PLWH. (A to C) Induction of unspliced (US) HIV-1 RNA in CD4^+^ T cells from 3 different HIV-1-infected individuals as presented in Fig. 5B and C shown as percent latency reversal relative to PMA/ionomycin treatment. Download FIG S7, TIF file, 1.6 MB. Copyright © 2021 Röling et al. 2021 Röling et al. https://creativecommons.org/licenses/by/4.0/ This content is distributed under the terms of the Creative Commons Attribution 4.0 International license.

Essential for the potential applicability of the novel LRA topiramate in the context of HIV-1 cure studies is the lack of off-target effects that eventually lead to significant cytotoxicity. Previously, we determined that topiramate, at working latency reversal concentrations, does not lead to significant global immune activation or cause apoptosis of primary CD4^+^ T cells (Fig. 4E to G). Additionally, to rigorously test for topiramate cytotoxicity in our system, we determined the 50% cytotoxic concentration (CC_50_) by measuring cell viability at different time points posttreatment (24 h, 48 h, and 72 h) in CD4^+^ T cells obtained from healthy donors using the alamarBlue viability assay (Fig. 5D) and determined a CC_50_ at 72 h of 49.27 μM (95% confidence interval [CI], 37.35 to 65.62) well above the lowest latency-reversing topiramate concentration (10 μM, Fig. 5B and C).

This observation advances topiramate as a prime clinically relevant candidate LRA that warrants further mechanistic investigation and inclusion in proof-of-concept clinical trials aiming to reverse latency and reduce the reservoir in PLWH.

## DISCUSSION

In search of potentially novel host factors and pathways that play a role in the maintenance of HIV-1 latency, we performed a two-color haploid genetic screen in latent HIV-1 infected KBM7 cells. An important advantage presented by this approach is that identification of putative functionally relevant candidate latency-promoting host target genes does not require *a priori* knowledge of the molecular determinants of latency and is thus completely unbiased. Additionally, gene-trap insertional mutagenesis has enabled identification of previously unappreciated latency genes and cellular pathways, which likely modulate latency not only via direct physical association with the HIV-1 promoter but also indirectly, through involvement in cellular signaling. We produced a list of 69 candidate genes and proceeded to validate 10 candidates, the depletion of which in various *in vitro* models of latency, including primary CD4^+^ T cell models, led to latency reversal.

The haploid KBM7 cell line, while a powerful system for GT-mediated forward genetic screens, is a myeloid cell line (34). Since HIV-1 can infect and establish latent infection in monocytes and macrophages (79, 80), a potentially important role for monocytes/macrophages as long-lived reservoirs for HIV-1 has been suggested. The candidates emerging from our screen therefore suggest a role for these genes in latency establishment in the myeloid lineage. As expected, candidate genes display robust expression not only in KBM7 cells but also in macrophages (Table 1). Moreover, shRNA depletion, as well as pharmacological inhibition of our most promising candidate target gene, GRIK5, in our myeloid cell-derived Hap-Lat cell line, indeed resulted in latency reversal (see Fig. S6C and D in the supplemental material). However, myeloid cells are not considered to be the prime source of the latent reservoir; this begs the question how relevant the myeloid nuclear environment may be for HIV-1 latency in lymphocytic cells. Nevertheless, when we tested a selected subset of the candidate genes by shRNA-mediated knockdown in a T-cell-derived model of HIV-1 latency, we found that knockdown of 67% of candidates (10 out of 15) led to latency reversal, which demonstrates the validity of our approach. Apart from the myeloid origin of our Hap-Lat model system, our study is also limited by the fact the we used a minimal HIV-1 construct that lacks Tat to conduct the GT screens and thus presents limits in identifying the full repertoires of genes associated with full-length viral latency. However, we believe that as a discovery tool, it provides us with a number of interesting new genes, previously not described to be involved in regulation of HIV-1. Several of the genes identified in our gene-trap screen have previously been implicated in HIV-1 susceptibility: 15 out of 69 genes are listed in the HIV-1 human interaction database (https://www.ncbi.nlm.nih.gov/genome/viruses/retroviruses/hiv-1/interactions/), a detailed database of all known interactions between HIV-1 and the human host. Furthermore, one of the candidates, IRF2BP2, is a potential target of HIV-1-associated nucleotide polymorphisms within a cluster of regulatory DNA elements (64) which loop to and potentially regulate the IRF2BP2 promoter in CD4^+^ T cells (81, 82). In the current study, we demonstrate that knockdown of IRF2BP2 results in latency reversal. IRF2BP2 has been shown to interact with NFAT1 and to repress transcriptional activity (83), providing a plausible mechanism for its role in latency maintenance.

Among the candidate gene list, we also identified CHD9, a member of the chromodomain helicase DNA binding (CHD) family of the ATP-dependent chromatin remodelers. Members of this family are involved in various cellular processes and in normal development and disease; however, CHD9 is one of the least-studied members. We found that CHD9 is associated with the latent HIV-1 5′ LTR and is displaced upon promoter activation by PMA stimulation, suggesting that it acts as a repressor of HIV-1 transcription. Indeed, depletion of CHD9 by shRNA-mediated knockdown led to derepression of HIV-1, as observed by an increase in the expression of the HIV-1 LTR-driven reporter GFP as well as the HIV-1 genes Gag, Pol, and Tat. Future studies will determine the mode of recruitment of CHD9 to the HIV-1 LTR and the molecular interplay between CHD9 and other chromatin remodeler and modifying complexes associated with the latent HIV-1 5′ LTR (4).

A large subset of the list of 69 candidate genes are noncoding RNAs (*n* = 22); upon closer inspection we found that 16 of those are at least in part oriented in an antisense direction with respect to known protein-coding genes. We currently do not know if these noncoding transcripts fulfill a biological function in maintenance of HIV-1 latency, directly through their transcripts, through regulatory effects on other (protein-coding) genes in *cis* or in *trans*, or through other effects, or if they represent mapping artifacts. Further investigation into these possibilities is ongoing.

An interesting observation emerging from our experimental setup is the appearance of a subpopulation of cells after multiple rounds of sorting that is more stable in its GFP expression. Thus, although the two populations overlap, one comprises stably reverted cells, while the other also includes more transiently reverted cells, which over time would return to a latent state. This subpopulation is also higher in mCherry expression, compared to the total double-positive population. The reversal of double-positive cells after sorting may reflect the intrinsically stochastic transcription of HIV-1 (84–86). Latent cell lines are notoriously sensitive to cellular stresses, which cause reactivation (87–89). It is, therefore, possible that the less-stable GFP-positive cells represent cells that are temporarily activated and which slowly revert to a latent state. Therefore, we focused our analysis and validation experiments mainly on the stable GFP-high population. Nevertheless, the few candidate genes unique to the total population that we tested in our shRNA knockdown validation experiment had a similar false-positive ratio (i.e., 33.3%) as we found for the candidate genes from the stable GFP-positive population.

For our analysis, we set a strict criterion of the LOF score being ≥3 in at least two biological replicates, although a cutoff at lower LOF scores of 2 or above has been used previously (90). We chose this stringent criterion to confidently identify 69 candidate genes. We believe, however, that our data set likely contains additional candidate genes that we miss due to the stringency applied. This is exemplified by our observation that STRING analysis indicates that many of the 598 protein-coding genes with an LOF score of 3 and higher functionally interact (Fig. S8). Moreover, 32% of these genes (195) appear in the HIV-1 human interaction database, pointing to their potential roles in HIV-1 biology. Importantly, proteins with well-described functions in HIV-1 biology, such as, e.g., IL-32 (LOF = 9.5) (64), RUNX1 (LOF = 12.6) (91), and SMARCA4/BRG1 (LOF = 8.7) (57), are detected with high LOF scores in at least one of our samples.

10.1128/mBio.02980-21.8 FIG S8 STRING analysis of protein coding candidate genes. STRING analysis indicates that many of the 598 protein-coding candidate genes with an LOF score of 3 and higher from the GFP^Total^ and the GFP^Stable^ populations functionally interact. Validated candidate genes are indicated in red. Circle size and green shade indicate the number of samples in which the gene-trap target gene is found. Circled genes are part of our candidate gene list. Background color indicates if the gene-trap target gene is found in the GFP^Total^, in the GFP^Stable^, or in both populations. Download FIG S8, TIF file, 1.1 MB. Copyright © 2021 Röling et al. 2021 Röling et al. https://creativecommons.org/licenses/by/4.0/ This content is distributed under the terms of the Creative Commons Attribution 4.0 International license.

Identification of functionally relevant molecular targets and novel compounds effective in latency reversal has proven to be an outstanding challenge in the field (92). Most clinically investigated LRAs have thus far failed to significantly impact the size of the latent reservoir in patients (92). From our list of validated host factors, we identified three druggable targets, ADK, NF1, and GRIK5. The ADK inhibitor 5-iodotubercidin displayed cytotoxicity in both J-Lat and primary models of HIV-1 latency (Fig. 4A, B, and E). Since knockdown of ADK did not lead to significant toxicity, off-target, pleiotropic effects of 5-iodotubercidin may underlie the observed T cell toxicity. 5-Iodotubercidin is the prototype nucleoside ADK inhibitor, with a relatively low reported CC_50_ of 1.12 μM (93). Novel nucleoside and nonnucleoside ADK inhibitors have been developed with more favorable pharmacological properties that would be attractive to pursue in the context of HIV-1 latency reversal in future studies (70). Of note, phosphorylation of the tricyclic nucleoside triciribine by ADK is required for its activity against HIV-1, which highlights the potential of interactions between LRAs and other HIV-1 therapeutics (94). Trametinib, a small-molecule inhibitor of NF1, reactivated HIV-1 in J-Lat cell models with modest effects on viability, which is in agreement with the high 48-h CC_50_ of 173.70 μM as reported for Vero cells (95) but nevertheless displayed significant toxicity in our primary cell models (Fig. 4A, B, and E). These results highlight the importance of interrogating the potential effectiveness of candidate LRAs in multiple models of HIV-1 latency to circumvent potential integration-site-mediated biases present in cell line models of latency and, most importantly, to confirm effectiveness in the more relevant primary CD4^+^ T cells obtained from cART-suppressed people living with HIV-1 (PLWH). Interestingly, trametinib has also been previously described to suppress HIV-1 viral replication via interference with the disassembly of the capsid core, an aspect which may also need to be considered in the context of its potential use as a latency reversal agent (96).

Our data especially highlight the FDA-approved GRIK5 inhibitor topiramate as a promising compound for latency reversal. Topiramate effectively reversed latency at low concentrations (10 to 20 μM) both in primary HIV-1-infected CD4^+^ T cells and in CD4^+^ T cells from three PLWH under suppressive antiretroviral therapy without inducing significant T cell activation or cytotoxicity (Fig. 4 and 5 and Fig. S6 and S7). Currently, topiramate is primarily used as an orally administered anticonvulsant or antiepileptic drug with a recommended daily dose for topiramate monotherapy in adults and children 10 years of age and older of 400 mg/day (resulting in a 22.7 μM peak serum concentration) but can even be tolerated as doses up to 2,400 mg (∼168 μM peak serum concentration) if titrated slowly (97). Therefore, the concentration of 10 to 20 μM topiramate we found to be sufficient for latency reversal falls well within the doses currently used in the clinic and safely tolerated by patients (97).

GRIK5 (glutamate ionotropic receptor kainate type subunit 5), primarily studied in neurons, is a subunit of the tetrameric kainate receptor (KAR), a subgroup of ionotropic glutamate receptors. GRIK5, together with GRIK4, binds glutamate, whereas subunits GRIK1 to -3 form functional ion channels (72). In B cells, KAR activation by glutamate increases ADAM10 levels, leading to increased B cell proliferation and immunoglobulin production (98). While the exact mechanism by which topiramate exerts anticonvulsant or antiepileptic properties is unclear, it has been shown to block voltage-dependent sodium and calcium channels (71, 99) and to inhibit the excitatory glutamate pathway and enhance inhibition by GABA (100). Interestingly, topiramate can induce cytochrome P450 family member CYP3A4 activity and potentially negatively affect the metabolism of many drugs (101), which should be taken into account when considering a potential therapeutic combination of LRAs in future studies.

In conclusion, we use a gene-trap screen in KBM7 Hap-Lat cells as a discovery tool to identify a number of interesting new genes, previously not described to be involved in regulation of HIV-1, and validate a subset in T cell line models and primary models of latency harboring the full-length virus. Topiramate, an FDA-approved inhibitor of the candidate GRIK5, is able to reverse latency in *ex vivo* treated CD4^+^ T cells from PLWH, is safe for use in patients, and is orally available and is therefore an attractive LRA candidate for direct inclusion in proof-of-concept clinical trials aiming to reverse latency and reduce the reservoir in PLWH.

## MATERIALS AND METHODS

### Cell culture.

KBM7 (a kind gift from Thijn Brummelkamp) and Hap-Lat (latent HIV-1 infected KBM7-derived cell line) cells were cultured in Iscove’s modified Dulbecco’s medium (IMDM) (ThermoFisher Scientific) supplemented with 10% fetal calf serum (FCS) and 2% penicillin-streptomycin (Pen/Strep). Haploidy of KBM7 cells was maintained by periodically sorting cells for size (5% smallest). Ploidy of cells was determined by propidium iodide staining using Jurkat cells as a diploid control.

### Establishment of haploid latent (Hap-Lat) HIV-1-infected cell lines.

We used a strategy described previously (56, 57) to generate latent HIV-1 infected KBM7 haploid cell lines. Minimal HIV-1 (LTR-GFP, HIV-1-658) (65) virus was produced by transfection of 293T cells in 15-cm culture dishes using polyethylenimine (PEI) (Sigma-Aldrich) with a mixture of 6.8 μg p658, 2 μg vesicular stomatitis virus G (VSVG), and 4.5 μg Gag-Pol plasmids. Haploid KBM7 cells were infected with HIV-1-derived virus at low MOI such that approximately 5 to 10% of cells became productively infected as determined by GFP expression. Five days after infection, the GFP-negative cell population harboring uninfected and potentially latently infected cells was sorted by flow cytometry-activated cell sorting (FACS) and stimulated with 350 nM suberoylanilide hydroxamic acid (SAHA) (Selleck Chem), and 5 ng/μl TNF-α (Sigma-Aldrich). Twenty-four hours poststimulation, GFP-positive cells were single cell sorted by FACS into 96-well plates. Clones were expanded and characterized for their basal GFP expression and their potential for reactivation. From the clonal cell lines generated, Hap-Lat was selected for low background and relative high reactivation upon stimulation (GFP negative under basal conditions but able to be induced to express GFP upon activation).

### Gene-trap virus production and mutagenesis of Hap-Lat cell lines.

We adapted a strategy described previously (35) to mutagenize Hap-Lat cells. Briefly, gene-trap virus was produced by transfection of HEK293FT cells in 15-cm culture dishes using 180 μl polyethylenimine (PEI) with a mixture of 8 μg pGT-mCherry, 2.1 μg pAdvantage, 3.1 μg cytomegalovirus (CMV)-VSVG, and 4.8 μg Gag-Pol plasmids in at total volume of 1 ml serum-free RPMI medium. After 12 h the growth medium was changed to fresh FCS and G418-supplemented RPMI. The virus was collected at 12, 24, 36, and 48 h. For the GT mutagenesis, 192 million Hap-Lat cells were preincubated with 100 μM deoxynucleoside triphosphates (dNTPs) (Invitrogen) 1 h before transduction. Cells were resuspended in 192 ml of undiluted GT virus. Spin infection was performed for 90 min at 1,500 rpm in 24-well plates containing 2 million cells per well and 8 μl protamine sulfate per ml. After 24 h cells were subjected to a second round of infection after which virus was removed, and cells were left to recover in supplemented IMDM. We estimate that this sequential infection resulted in 1 to 4 GT integrations per cell, with the majority of cells containing one integration (data not shown). A first round of sorting was performed 8 days after the second infection. Subsequently, between 500,000 and 1 million GFP-mCherry double-positive cells were sorted each day for 5 consecutive days using two cell sorters (BD FACSAria II SORP and BD FACSAria III). Sorted cells were pooled, left to recover, and expanded for the next round of sorting. A second round of sorting was performed 17 days after the second infection. Between 2.6 and 3 million cells were collected each day for 3 consecutive days using two sorters. These cells were pooled and left to recover and expand for a third round of sorting on day 22 after the second infection, yielding 6.1 million cells from 1-day sorting on two machines. On day 29 after the second infection a fourth round of sorting was performed, yielding 7.2 million cells from 1-day sorting on two machines. After the cells of the fourth round of sorting were left to recover and expand, a subpopulation of highly mCherry and GFP double-positive cells were apparent. In a final fifth round of sorting the total GFP-positive mCherry-positive population (10.1 million cells) was sorted as soon as enough cells were available (day 33 after the second infection) while the GFP-mCherry-positive stable subpopulation (5.9 million cells) was cultured for several more days and sorted (>>35 days after the second infection) (see Fig. S1J in the supplemental material). After recovery, genomic DNA was isolated from the sorted cell populations, as well as from a population of mutagenized unsorted cells.

### Mapping insertion sites through inverse PCR.

A previously described inverse PCR protocol was adapted to determine host sequences flanking the proviral insertion sites (35). Briefly, genomic DNA was isolated from 5 million cells using the DNeasy kit (Qiagen). Four micrograms of genomic DNA (gDNA) was digested with NlaIII or MseI. After PCR spin column purification (Qiagen), 50 μl of eluted digested DNA was ligated using T4 DNA ligase (Roche) in a volume of 2 ml. The reaction mix was purified using spin columns and used as the template in a PCR with primers annealing to internal sequences in the gene-trap vector (5′-CTGCAGCATCGTTCTGTGTT-3′ and 5′-TCTCCAAATCTCGGTGGAAC-3′). The resulting PCR products were used for library preparation.

### High-throughput sequencing and identification of integration sites.

Sequencing libraries were created using the Ion Plus fragment library kit (ThermoFisher Scientific) according to manufacturer’s instructions, with minor modifications: briefly, 15 ng of the PCR products was diluted with double-distilled water (ddH_2_O) to a final volume of 39 μl. The samples were end repaired, adaptor ligated, and amplified; this was followed by 2 rounds of purification using Agencourt AMPure XP beads. Library qualities and quantities were assessed by Bioanalyzer, using the DNA High-Sensitivity kit (Agilent Technologies). The quantified libraries were pooled in 10-plex, at a final concentration of 40 pM. Templating, enrichment, and chip loading were performed on an Ion Proton Chef system using the Ion PI Hi-Q Chef kits (ThermoFisher Scientific); sequencing was performed on an Ion Proton PI V3 chip, with the Ion PI Hi-Q Sequencing 200 kit, on an Ion Proton instrument (ThermoFisher Scientific), according to manufacturer’s instructions.

The resulting FASTQ files were processed to remove the gene-trap vector primers. To this end, we used R/Bioconductor packages ShortRead (102) and Biostrings to match primer sequences within the sequenced reads, allowing for two mismatches at maximum for each primer sequence. The identified primers were trimmed, and reads shorter than 15 bp were discarded. The remaining reads were aligned to the human reference genome version hg19 retrieved from the Illumina iGenomes repository, using bwa mem. The produced aligned reads (BAM files) were then subjected to integration site identification. To this end, we reimplemented the HaSAPPy algorithm (62) in R. The reimplemented version operates on BAM instead of SAM files and was enriched with additional noise-filtering steps, which improved the overall process of detecting integration sites using the Local Outlier Method (LOF), as described in the original HaSAPPy implementation. Specifically, we added attributes for (i) filtering out read artifacts from introns, based on the overall intronic read distribution for each sample; (ii) selecting the fraction of neighboring (independent insertions, disrupting insertions, bias) triplets for LOF analysis; and (iii) library normalization across samples. After the quality control steps, our HaSAPPY reimplementation assigns a Local Outlier Factor (LOF) score to each gene in a sample based on a triplet score derived from the number of putative GT integrations in the sample compared to the reference, additionally taking into account the potential bias arising from unbalanced sense and antisense read numbers. LOF is a metric to indicate to what extent a vector measurement deviates from a population of vectors with the same properties. In our case, a triplet vector corresponds to a gene and LOF measures the deviation from the respective population, i.e., a gene is an outlier based on a distribution of vectorized scores, such as the aforementioned triplet. The reimplemented version can be found in https://github.com/pmoulos/ngs-stone-age/blob/master/R/hasar.R.

### shRNA knockdown of candidate genes in A2 and 11.1 cell lines.

Predesigned shRNA sequences (Mission shRNA library [Sigma]; Table 2) were purchased as bacterial glycerol stocks from the Erasmus Center for Biomics. Virus was produced as follows. HEK293T cells at 3 × 10^5^/ml were plated in a 10-cm dish 1 day before transfection and transfected using PEI with 4.5 μg of pCMVΔR8.9 (envelope), 2 μg of pCMV-VSV-G (packaging), and 6 μg of shRNA vector. The transfection mixture was removed after 12 h and replaced with fresh RPMI medium containing 10% fetal bovine serum (FBS). Virus-containing supernatant was collected at 36, 48, 60, and 72 h posttransfection. Jurkat, J-Lat A2 (LTR-Tat-IRES-GFP), and J-Lat 11.1 (integrated full-length HIV-1 genome mutated in *env* gene and GFP replacing Nef) cells were cultured in RPMI 1640 medium (Sigma) supplemented with 10% FBS and 100 μg/ml penicillin-streptomycin at 37°C in a humidified 95% air-5% CO_2_ atmosphere. A2 and 11.1 cell lines were infected by adding 500 μl of filtered lentivirus to 2.5 ml of cell culture. After 48 h, medium was refreshed and cells were puromycin selected. At 14 days after infection, RNA was harvested to determine knockdown of genes by qPCR and reactivation of latent HIV-1 was measured by flow cytometry and RT-qPCR for Gag, Pol, and Tat.

**TABLE 2:** List of Mission shRNA clones

Recombinant DNA		
sh ADK	Mission shRNA library (Sigma)	TRCN000011535
sh GRIK5	Mission shRNA library (Sigma)	TRCN000011537
sh SPN	Mission shRNA library (Sigma)	TRCN000011539
sh Control	Mission shRNA library (Sigma)	SHC002
sh CMSS1	Mission shRNA library (Sigma)	TRCN000011541
sh EVL	Mission shRNA library (Sigma)	TRCN000011542
sh COPS5	Mission shRNA library (Sigma)	TRCN000011543
sh EXOSC8	Mission shRNA library (Sigma)	TRCN000011544
sh SLC7A6	Mission shRNA library (Sigma)	TRCN000011545
sh USP15	Mission shRNA library (Sigma)	TRCN000011547
sh NF1	Mission shRNA library (Sigma)	TRCN000011638
sh IRF2BP2	Mission shRNA library (Sigma)	TRCN000011635
sh RHOF	Mission shRNA library (Sigma)	TRCN000011642
sh EVI2B	Mission shRNA library (Sigma)	TRCN000011644
sh CHD9	Mission shRNA library (Sigma)	TRCN0000230236
sh FAM19A	Mission shRNA library (Sigma)	TRCN000011641

### Flow cytometry for GFP expression in the J-Lat cell lines.

Cells were collected in phosphate-buffered saline (PBS). GFP fluorescent signal indicating latency reversal was monitored using an LSRFortessa (BD Biosciences). Viability was determined using the forward scatter area versus side scatter area profiles (FSC-A and SSC-A, respectively). Data were analyzed using FlowJo software (version 9.7.4; TreeStar).

### Total RNA isolation and quantitative RT-PCR (RT-qPCR).

Total RNA was isolated from transduced A2 and 11.1 cells using TRIzol (ThermoFisher) on day 14 after infection using the total RNA Zol Out kit (A&A Biotechnology), and residual genomic DNA was digested with DNase I (Life Technologies). cDNA was synthesized using Superscript II reverse transcriptase (Life Technologies) using oligo(dT) primers or random primers (for Gag, Pol, and Tat). RT-quantitative PCRs (RT-qPCRs) were performed on a CFX Connect real-time PCR detection system thermocycler (Bio-Rad) using GoTaq qPCR master mix (Promega) (3 min at 95°C, followed by 40 cycles of 95°C for 10 s and 60°C for 30 s). Melting-curve analysis was performed to assess specificity of RT-qPCR products. Primers used for real-time PCR are listed in Table 3. Expression data were calculated using the threshold cycle (2^−ΔΔ^*^CT^*) method (103). β-2-Microglobulin (B2M) and glyceraldehyde-3-phosphate dehydrogenase (GAPDH) were used as housekeeping genes for the analysis.

**TABLE 3:** List of qPCR primers

List of RT-qPCR primers		
Name of gene	Forward primer	Reverse primer
ADK	GACACAAGCCCTGCCAAAGAT	TCAGAGACCAGTTGAGACAGAA
CMSS1	GCCAATGATTTGACTCACAGTCT	CTGAATGCTGTCATCGACCTAAT
IRF2BP2	CCCATGACTCCTACATCCTCTT	GAGGGCGGACTGTTGCTATTC
NF1	AGATGAAACGATGCTGGTCAAA	CCTGTAACCTGGTAGAAATGCGA
CHD9	GAAATGCTAGAAAGGTTGGAGG	CGGGACCAGTGAGAATACGTT
FAM19A	TGTTAAAACGGGAACTTGTGAGG	AAGCATCCACACATGATGGAG
SCN9A	ATTCGTGGCTCCTTGTTTTCTG	CTACTGGCTTGGCTGATGTTAC
RHOF	CCCCATCGGTGTTCGAGAAG	GGCCGTGTCGTAGAGGTTC
GRIK5	CCACCGTGAGCCATATCTGTG	CGCGAAGCGAAGGTACTGAA
USP15	CGACGCTGCTCAAAACCTC	TCCCATCTGGTATTTGTCCCAA
EVL	CTTCCGTGATGGTCTACGATG	TGCAACTTGACTCCAACGACT
EXOSC8	CGGTTCAATTAGTACCGCAGAT	ACGTATCCTTTATCAGGGGCAT
COPS5	TGGGTCTGATGCTAGGAAAGG	CTATGATACCACCCGATTGCATT
SLC7A6	CGGGCTTTCAGTTGTTAGACC	ACACAGTCAATCGCTCACGG
EVI2B	ACCAACACAATTCAGCGACAC	GTTGTAGGCAAGTGGTTGTCC
SPN	GCTGGTGGTAAGCCCAGAC	GGCTCGCTAGTAGAGACCAAA
POL	GGTTTATTACAGGGACAGCAGAGA	ACCTGCCATCTGTTTTCCATA
GAG	AGTAGTGTGTGCCCGTCTGT	TCGCTTTCAGGTCCCTGTTCG
TAT	CAAAAGCCTTAGGCATCTCCT	CCACCTTCTTCTTCGATTCCT
β2Microglobulin	AGCGTACTCCAAAGATTCAGGTT	ATGATGCTGCTTACATGTCTCGAT
GAPDH	CAAGAAGGTGGTGAAGCAG	GCCAAATTCGTTGTCATACC
List of ChIP-qPCR primers		
Amplicon	Forward primer	Reverse primer
HIV-1Nuc0 (5′ + 3′ LTR)	CCACACACAAGGCTACTTCC	AACTGGTACTAGCTTGTAGCAC
HIV-1 HSS1 (5′ + 3′ LTR)	TGTGAGCCTGCATGGGATGG	GAAAGTCCCCAGCGGAAAGT
HIV-1Nuc1/HSS2 (5′ + 3′ LTR)	CGTCTGTTGTGTGACTCTGGT	TCGAGAGAGCTCCTCTGGTT
HIV-1HSS2/Nuc2 (5′ LTR)	GCCCGAACAGGGACTTGAAA	TTGGCGTACTCACCAGTCG
HIV-1Nuc2/Gag (5′ LTR)	GGTGCGAGAGCGTCAGTAT	AGCTCCCTGCTTGCCCATA
Hk2	GCCGACTCTTGTATTGCCTG	TATTGTAGCACGGCCGGAAA
GFP (HIV-1)	GGAGTGGTCCCAGTTCTTGTTG	ACAGGTAGCTTCCCAGTAGTGC

### Isolation and *ex vivo* infection of primary CD4^+^ T cells.

An HIV-1 latency *ex vivo* model was generated based on the Lassen and Greene method by spinoculation (90 min at 1,200 × *g*), washed in PBS, and cultured in RPMI 1640 containing 10% FBS, 100 μg/ml penicillin-streptomycin, interleukin-2 (IL-2) (5 ng/ml), and the antiretroviral drug saquinavir mesylate (5 μM) (77). HEK293T cells were transfected with HXB2 Env and pNL4.3.Luc.R-E- plasmids using PEI (polyethylenimine) as described above. Supernatants containing pseudovirus particles were collected at 24, 48, and 72 h posttransfection. Peripheral blood mononuclear cells (PBMCs) were isolated by Ficoll density gradient sedimentation of buffy coats from healthy donors. Total CD4^+^ T cells were separated by negative selection using RosetteSep human CD4^+^ T cell enrichment cocktail (StemCell Technologies). Primary CD4^+^ T cells were plated at a concentration of 1.5 × 10^6^ cells/ml, left overnight for recovery, and infected. Two days after infection, cells were collected, washed once with PBS, and treated with 5-iodotubercidin, topiramate, or trametinib. Cells were collected 48 h after treatment and washed once in PBS, and luciferase activity was measured using the luciferase assay system (Promega).

### Flow cytometry for T cell activation and toxicity assay.

Primary CD4^+^ T cells isolated from buffy coats of healthy volunteers were treated with different concentration of 5-iodotubercidin, topiramate, and trametinib and PMA/ionomycin as a positive control. After 24 and 48 h, cells were stained with annexin V to examine the percentage of cells undergoing apoptosis and with the surface receptor CD69 and CD25 to measure T cell activation. For staining, 10^6^ cells were washed with PBS supplemented with 3% FBS and 2.5 mM CaCl_2_ followed by staining with annexin V-phycoerythrin (PE) (Becton Dickinson), CD69-fluorescein isothiocyanate (FITC) (eBioscience), and CD25-allophycocyanin (APC) (eBioscience) for 20 min at 4°C in the presence of 2.5 mM CaCl_2_. Cells were then washed with PBS-FBS-CaCl_2_ and analyzed by flow cytometry. Between 2 × 10^5^ and 4 × 10^5^ events were collected per sample within 3 h after staining on an LSRFortessa (BD Biosciences, 4 lasers, 18 parameters) and analyzed using FlowJo software (version 9.7.4, TreeStar).

### CC_50_ viability assay.

Primary CD4^+^ T cells from healthy donors were isolated from PBMCs as described above, incubated in RPMI medium supplemented with 10% fetal bovine serum and 1% penicillin-streptomycin (RPMI 10), and left to rest overnight. One million CD4^+^ T cells were plated in duplicate and treated with increasing concentrations of topiramate, 5-iodotubercidin, and trametinib as indicated. At different time points posttreatment (24 h, 48 h, and 72 h), cell viability was measured using the alamarBlue viability assay (alamarBlue; Invitrogen DAL1025; 1:10 in DM) according to the manufacturer’s instructions. Briefly, medium containing the treatment was removed from the wells, and 10% alamarBlue was dissolved in RPMI 10 and incubated for 2 h at 37°C. Absorbance was measured at 600 nm, and the readings were normalized to untreated control. The 50% cytotoxic concentration (CC_50_) value was defined as the concentration of the compound that reduced viability of the cells by 50% compared with the nontreated cell control at 72 h posttreatment. The CC_50_ value was calculated based on a nonlinear fit model using GraphPad v.8.

### Western blotting.

Cells (10 × 10^6^) were lysed for 30 min on ice with 200 μl lysis buffer (150 mM NaCl, 30 mM Tris [pH 7.5], 1 mM EDTA, 1% Triton X-100, 10% glycerol, 0.5 mM dithiothreitol [DTT], protease inhibitor cocktail tablets [EDTA-free] [Roche]). Cell lysates were clarified by centrifugation (14,000 rpm for 30 min at 4°C), mixed with 4× sodium dodecyl sulfate (SDS) loading buffer containing 0.1 M DTT, and boiled at 95°C for 5 min. Samples were run in a 10% SDS-polyacrylamide gel at 100 V. The proteins were transferred to polyvinylidene difluoride (PVDF) membranes, and the membranes were probed with anti-CHD9 (13402-1-AP; Proteintech) and anti-α-tubulin (ab6160; Abcam). The next day, blots were incubated with horseradish peroxidase (HRP)-conjugated secondary antibody, and proteins were imaged by chemical luminescence using Super Signal West Pico (Thermo Scientific).

### Chromatin immunoprecipitation (ChIP) and qPCR.

Approximately 50 to 100 million J-Lat 11.1 cells per condition were collected and cross-linked with 1% formaldehyde (Polysciences Inc.) in 40 ml PBS, supplemented with 1 mM MgCl_2_ and 1 mM CaCl_2_, at room temperature (RT) for 30 min with 15-rpm vertical rotation. The reaction was quenched with 125 mM glycine. Cross-linked cells were pelleted at 800 relative centrifugal force (rcf) for 5 min at RT and subjected to chromatin enrichment as described previously (104). Briefly, the pellets were resuspended in 2 ml ChIP incubation buffer (1% SDS, 1% Triton X-100, 150 mM NaCl, 1 mM EDTA, pH 8.0, 0.5 mM EGTA, 20 mM HEPES, pH 7.6, protease inhibitor cocktail tablets [EDTA-free; Roche]) containing 1% SDS and sonicated (30-s ON/30-s OFF intervals; Diogenode Bioruptor Plus) to obtain chromatin fragments between 300 and 500 bp. Sonicated chromatin was spun at 20,817 rcf at 4°C for 30 min and diluted 10 times in ChIP incubation buffer without SDS. Diluted chromatin corresponding to 30 to 40 million cells was precleared overnight at 4°C with vertical rotation at 15 rpm with 80 μl of protein A Sepharose 4 Fast Flow (GE Healthcare) beads and immunoprecipitated overnight with 40 μl of beads and 5 μg of antibody (13402-1-AP; Proteintech). Samples were washed two times per each buffer (5 min, 15-rpm vertical rotation) with buffer 1 (0.1% SDS, 0.1% deoxycholate [DOC], 1% Triton X-100, 150 mM NaCl, 1 mM EDTA, pH 8.0, 0.5 mM EGTA, 20 mM HEPES, pH 8.0.), buffer 2 (0.1% SDS, 0.1% DOC, 1% Triton X-100, 500 mM NaCl, 1 mM EDTA, pH 8.0, 0.5 mM EGTA, 20 mM HEPES, pH 8.0), buffer 3 (0.25 M LiCl, 0.5% DOC, 0.5% NP-40, 1 mM EDTA, pH 8.0, 0.5 mM EGTA, pH 8.0, 20 mM HEPES, pH 8.0), and buffer 4 (1 mM EDTA, pH 8.0, 0.5 mM EGTA, pH 8.0, 20 mM HEPES, pH 8.0) to remove unspecific binding. Finally, each sample was eluted in 400 μl elution buffer (1% SDS, 0.1 M NaHCO_3_), de-cross-linked overnight at 65°C in the presence of 200 mM NaCl, phenol-chloroform extracted, ethanol precipitated, and subjected to qPCR analysis using the primer sets summarized in Table 3.

### CA US HIV-1 RNA quantification.

CD4^+^ T cells from PLWH were isolated from frozen PBMCs by the EasySep CD4^+^ T cell isolation kit following the manufacturer’s instructions and were left to rest overnight. Between 1.5 and 2 million CD4^+^ cells were plated in duplicate in RPMI medium containing 10% FBS, 1% penicillin-streptomycin, and 30 mM raltegravir and treated with topiramate at the indicated concentrations for 24 h. Cells were then lysed with TRI reagent, and total RNA was isolated by chloroform precipitation followed by cDNA synthesis using Superscript II reverse transcriptase following the manufacturer’s instructions. Change in cell-associated (CA) unspliced HIV-1 RNA copies was determined by nested qPCR as described before (105). Briefly, the first round of amplification was performed in a final volume of 25 μl using 10 μl of cDNA, 2.5 μl of 10× PCR buffer (Life Technologies), 1 μl of 50 mM MgCl_2_ (Life Technologies), 1 μl of 10 mM dNTPs (Life Technologies), 0.075 μl of 100 μM Gag forward primer, 0.075 μl of 100 μM SK437 reverse primer, and 0.2 μl Platinum *Taq* polymerase (Life Technologies). The second round of amplification was performed in a final volume of 25 μl using 2 μl of preamplified cDNA, 2.5 μl of 10× PCR buffer (Life Technologies), 1 μl of 50 mM MgCl_2_ (Life Technologies), 1 μl of 10 mM dNTPs (Life Technologies), 0.05 μl of 100 μM Gag forward primer, 0.05 μl of 100 μM Gag reverse primer, 0.075 μl of 50 μM Gag probe, and 0.2 μl Platinum *Taq* polymerase. The absolute number of gag copies in the PCR was calculated using a standard curve ranging from 8 to 512 copies of a plasmid containing the full-length HIV-1 genome (pNL4.3.Luc.R-E-). The amount of HIV-1 cellularly associated RNA was expressed as number of copies/500 ng of input RNA in reverse transcription.

The study was conducted according to the ethical principles of the Declaration of Helsinki. All patients involved in the study gave their written informed consent. The study protocol and any amendment were approved by The Netherlands Medical Ethics Committee (MEC-2012-583).

### Data availability.

All sequencing data have been deposited to the Gene Expression Omnibus (GEO) database with accession code GSE189314.
